# Supplementary material for: High-throughput sequencing of multiple amplicons for barcoding and integrative taxonomy
Source: Sci Rep. 2017 Feb 6;7:41948. doi: 10.1038/srep41948 (PMC5292727; doi:10.1038/srep41948)
Supplement: Supplementary Dataset 1 [file srep41948-s1.pdf]

High throughput sequencing of multiple amplicons for barcoding and integrative taxonomy

Perrine Cruaud[1], Jean-Yves Rasplus[1], Lillian Jennifer Rodriguez[12] and Astrid Cruaud[1]*

[1] INRA, UMR1062 CBGP, F-34988 Montferrier-sur-Lez, France

[2] University of the Philippines - College of Science, Institute of Biology, Quezon City, Philippines.

Supplementary data

Fig S1. RAxML tree for the *COI* data set (Miseq+Sanger) (BP : 1000 replicates).
Prefix : I = Illumina, S = Sanger; Suffix : cl1 to cln = cluster ranking as return by the first step of the workflow (from read filtering to clustering), cl1 = the cluster that contains the largest proportion of reads/sequences. Problematic species as discussed in text are highlighted in blue (specimens for which two clusters of sequences were retained) and in green (species recovered paraphyletic or clustered into divergent groups of sequences (>7%)).

Fig S2. RAxML tree for the *Cytb* data set (Miseq+Sanger) (BP : 1000 replicates).
Prefix : I = Illumina, S = Sanger; Suffix : cl1 to cln = cluster ranking as return by the first step of the workflow (from read filtering to clustering), cl1 = the cluster that contains the largest proportion of reads/sequences. Problematic species as discussed in text are highlighted in blue (specimens for which two clusters of sequences were retained) and in green (species recovered paraphyletic or clustered into divergent groups of sequences (>7%)).

Fig S3. RAxML tree for the *EF* data set (Miseq+Sanger) (BP : 1000 replicates).
Prefix : I = Illumina, S = Sanger.

Fig S1. RAxML tree for the COI data set (Miseq+Sanger) (BP : 1000 replicates).

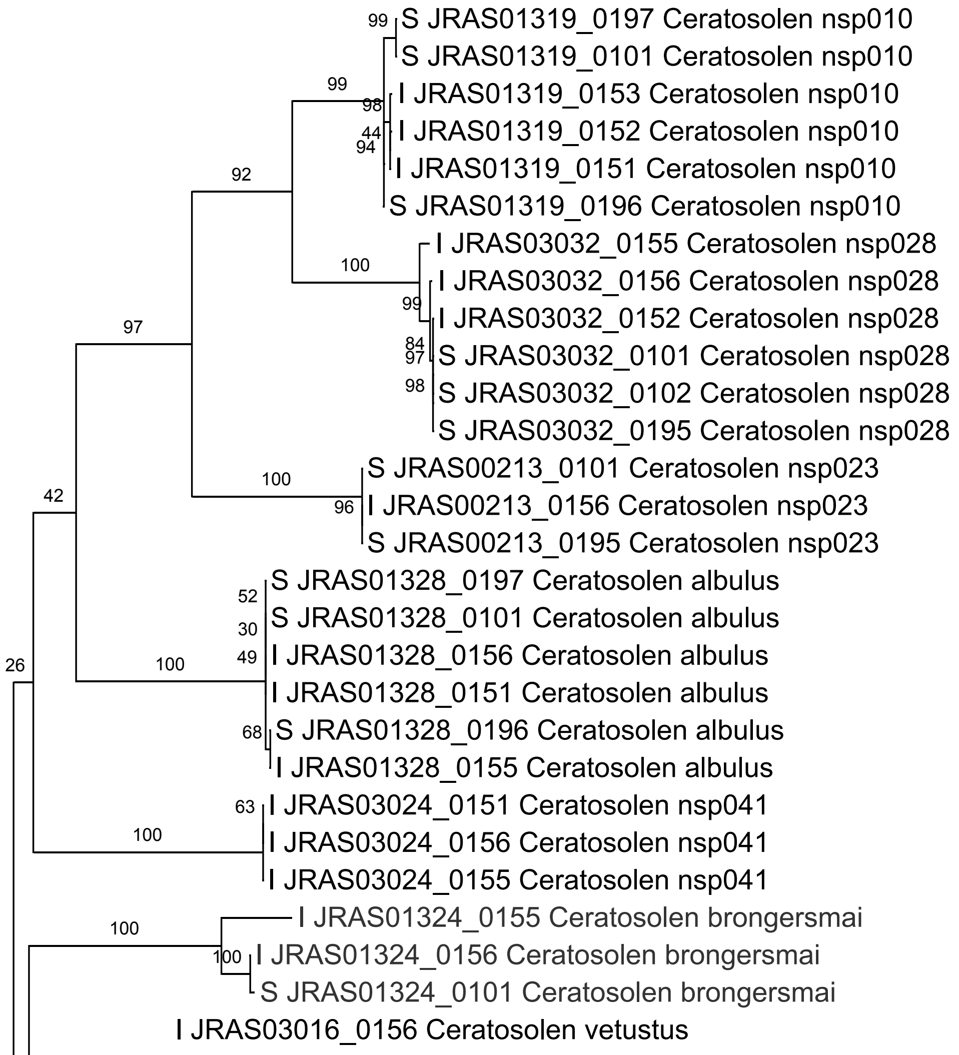

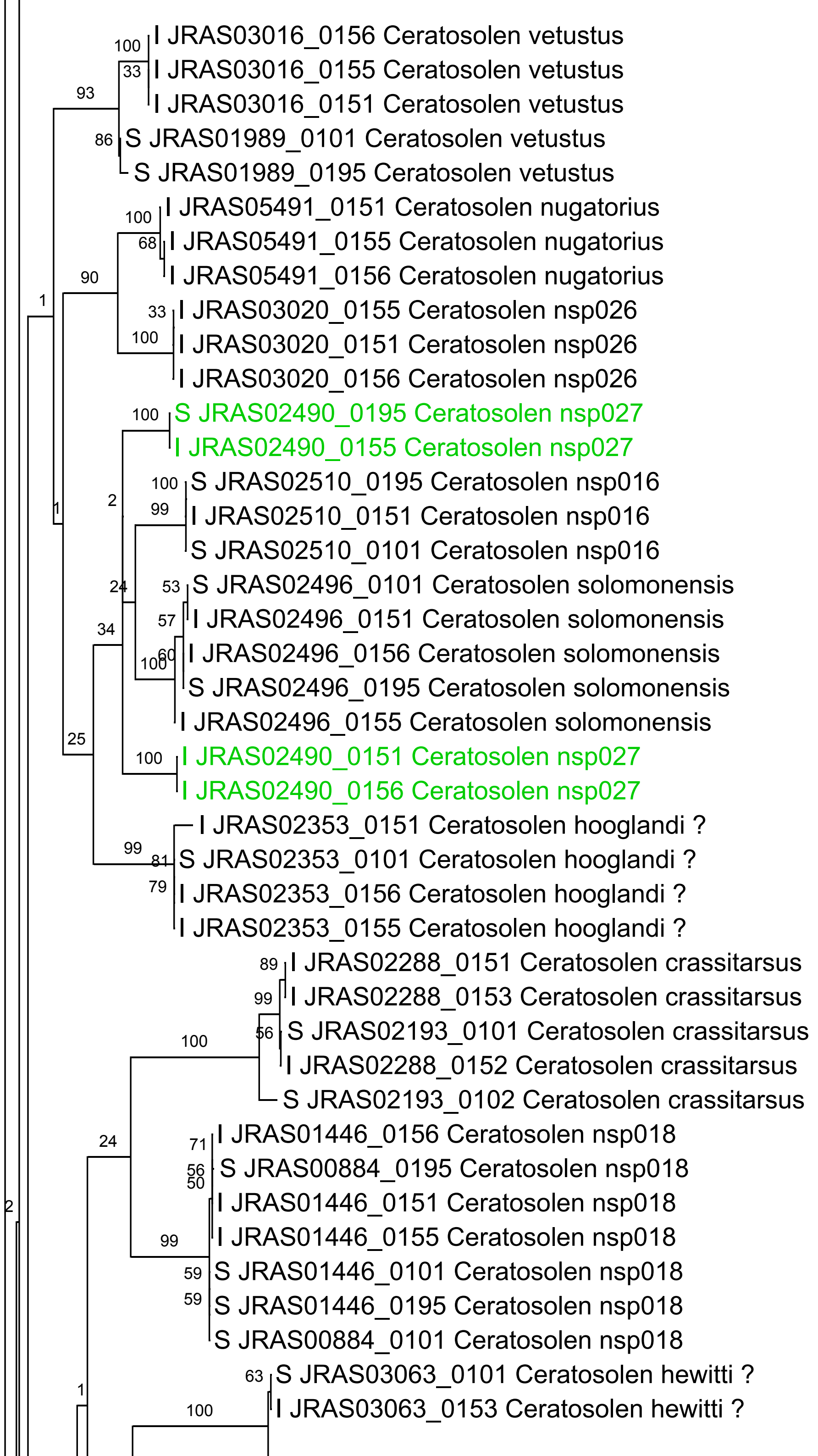
I JRAS03016_0156 Ceratosolen vetustus
I JRAS03016_0155 Ceratosolen vetustus
I JRAS03016_0151 Ceratosolen vetustus
S JRAS01989_0101 Ceratosolen vetustus
S JRAS01989_0195 Ceratosolen vetustus
I JRAS05491_0151 Ceratosolen nugatorius
I JRAS05491_0155 Ceratosolen nugatorius
I JRAS05491_0156 Ceratosolen nugatorius
I JRAS03020_0155 Ceratosolen nsp026
I JRAS03020_0151 Ceratosolen nsp026
I JRAS03020_0156 Ceratosolen nsp026
S JRAS02490_0195 Ceratosolen nsp027
I JRAS02490_0155 Ceratosolen nsp027
S JRAS02510_0195 Ceratosolen nsp016
I JRAS02510_0151 Ceratosolen nsp016
S JRAS02510_0101 Ceratosolen nsp016
S JRAS02496_0101 Ceratosolen solomonensis
I JRAS02496_0151 Ceratosolen solomonensis
I JRAS02496_0156 Ceratosolen solomonensis
S JRAS02496_0195 Ceratosolen solomonensis
I JRAS02496_0155 Ceratosolen solomonensis
I JRAS02490_0151 Ceratosolen nsp027
I JRAS02490_0156 Ceratosolen nsp027
I JRAS02353_0151 Ceratosolen hooglandi ?
S JRAS02353_0101 Ceratosolen hooglandi ?
I JRAS02353_0156 Ceratosolen hooglandi ?
I JRAS02353_0155 Ceratosolen hooglandi ?
I JRAS02288_0151 Ceratosolen crassitarsus
I JRAS02288_0153 Ceratosolen crassitarsus
S JRAS02193_0101 Ceratosolen crassitarsus
I JRAS02288_0152 Ceratosolen crassitarsus
S JRAS02193_0102 Ceratosolen crassitarsus
I JRAS01446_0156 Ceratosolen nsp018
S JRAS00884_0195 Ceratosolen nsp018
I JRAS01446_0151 Ceratosolen nsp018
I JRAS01446_0155 Ceratosolen nsp018
S JRAS01446_0101 Ceratosolen nsp018
S JRAS01446_0195 Ceratosolen nsp018
S JRAS00884_0101 Ceratosolen nsp018
S JRAS03063_0101 Ceratosolen hewitti ?
I JRAS03063_0153 Ceratosolen hewitti ?

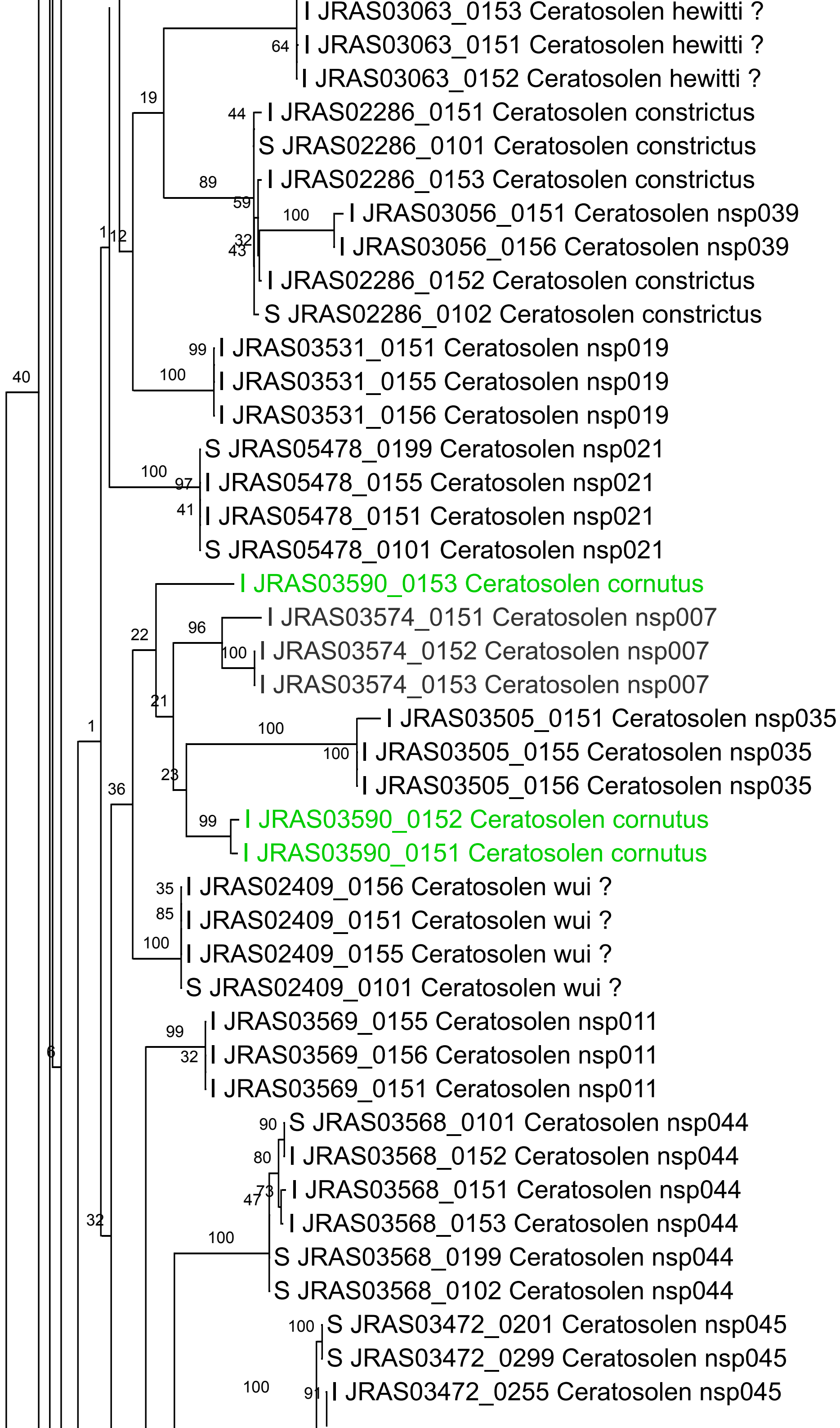
I JRAS03063_0153 Ceratosolen hewitti ?
I JRAS03063_0151 Ceratosolen hewitti ?
I JRAS03063_0152 Ceratosolen hewitti ?
I JRAS02286_0151 Ceratosolen constrictus
S JRAS02286_0101 Ceratosolen constrictus
I JRAS02286_0153 Ceratosolen constrictus
I JRAS03056_0151 Ceratosolen nsp039
I JRAS03056_0156 Ceratosolen nsp039
I JRAS02286_0152 Ceratosolen constrictus
S JRAS02286_0102 Ceratosolen constrictus
I JRAS03531_0151 Ceratosolen nsp019
I JRAS03531_0155 Ceratosolen nsp019
I JRAS03531_0156 Ceratosolen nsp019
S JRAS05478_0199 Ceratosolen nsp021
I JRAS05478_0155 Ceratosolen nsp021
I JRAS05478_0151 Ceratosolen nsp021
S JRAS05478_0101 Ceratosolen nsp021
I JRAS03590_0153 Ceratosolen cornutus
I JRAS03574_0151 Ceratosolen nsp007
I JRAS03574_0152 Ceratosolen nsp007
I JRAS03574_0153 Ceratosolen nsp007
I JRAS03505_0151 Ceratosolen nsp035
I JRAS03505_0155 Ceratosolen nsp035
I JRAS03505_0156 Ceratosolen nsp035
I JRAS03590_0152 Ceratosolen cornutus
I JRAS03590_0151 Ceratosolen cornutus
I JRAS02409_0156 Ceratosolen wui ?
I JRAS02409_0151 Ceratosolen wui ?
I JRAS02409_0155 Ceratosolen wui ?
S JRAS02409_0101 Ceratosolen wui ?
I JRAS03569_0155 Ceratosolen nsp011
I JRAS03569_0156 Ceratosolen nsp011
I JRAS03569_0151 Ceratosolen nsp011
S JRAS03568_0101 Ceratosolen nsp044
I JRAS03568_0152 Ceratosolen nsp044
I JRAS03568_0151 Ceratosolen nsp044
I JRAS03568_0153 Ceratosolen nsp044
S JRAS03568_0199 Ceratosolen nsp044
S JRAS03568_0102 Ceratosolen nsp044
S JRAS03472_0201 Ceratosolen nsp045
S JRAS03472_0299 Ceratosolen nsp045
I JRAS03472_0255 Ceratosolen nsp045

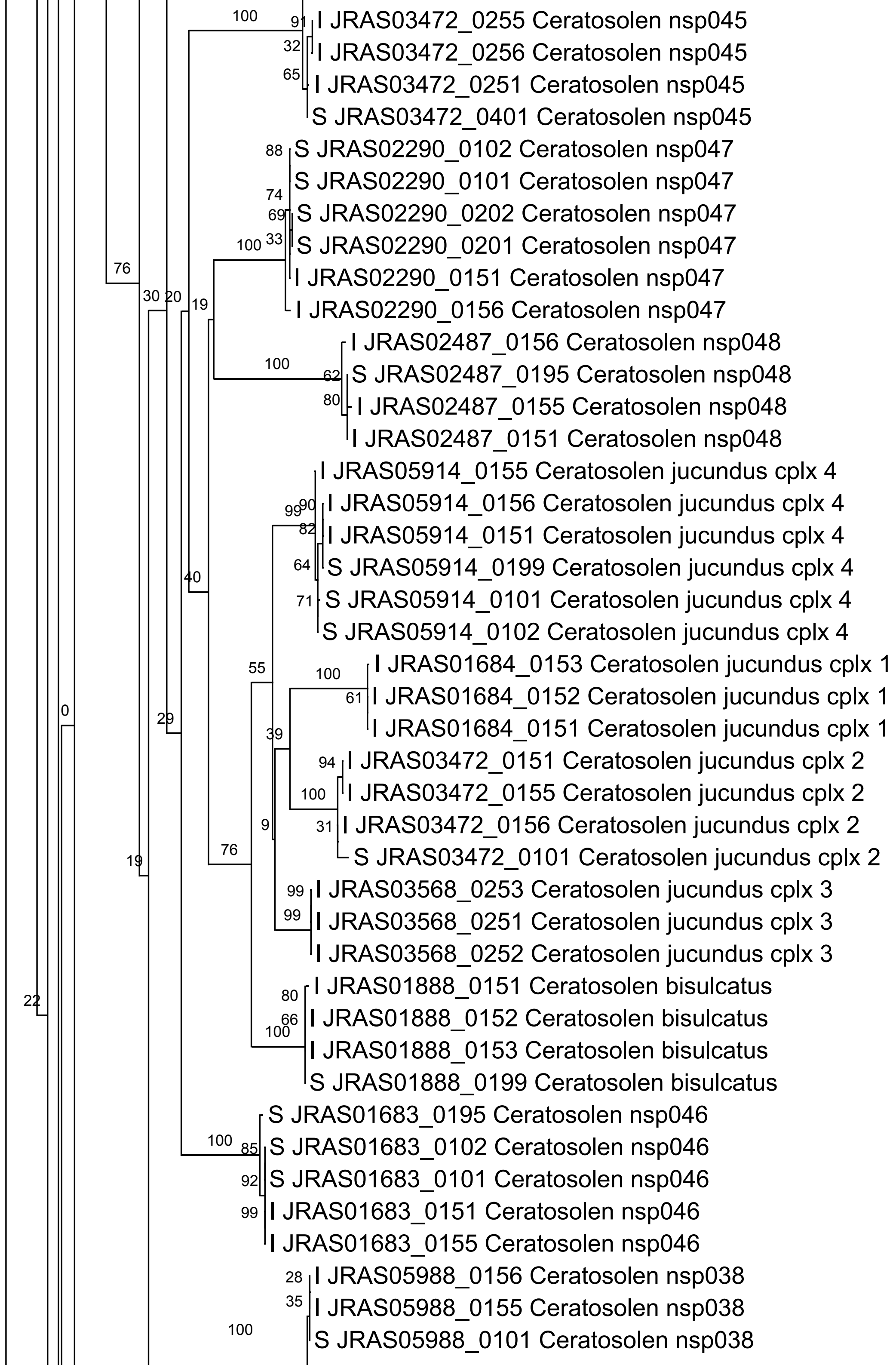
I JRAS03472_0255 Ceratosolen nsp045
I JRAS03472_0256 Ceratosolen nsp045
I JRAS03472_0251 Ceratosolen nsp045
S JRAS03472_0401 Ceratosolen nsp045
S JRAS02290_0102 Ceratosolen nsp047
S JRAS02290_0101 Ceratosolen nsp047
S JRAS02290_0202 Ceratosolen nsp047
S JRAS02290_0201 Ceratosolen nsp047
I JRAS02290_0151 Ceratosolen nsp047
I JRAS02290_0156 Ceratosolen nsp047
I JRAS02487_0156 Ceratosolen nsp048
S JRAS02487_0195 Ceratosolen nsp048
I JRAS02487_0155 Ceratosolen nsp048
I JRAS02487_0151 Ceratosolen nsp048
I JRAS05914_0155 Ceratosolen jucundus cplx 4
I JRAS05914_0156 Ceratosolen jucundus cplx 4
I JRAS05914_0151 Ceratosolen jucundus cplx 4
S JRAS05914_0199 Ceratosolen jucundus cplx 4
S JRAS05914_0101 Ceratosolen jucundus cplx 4
S JRAS05914_0102 Ceratosolen jucundus cplx 4
I JRAS01684_0153 Ceratosolen jucundus cplx 1
I JRAS01684_0152 Ceratosolen jucundus cplx 1
I JRAS01684_0151 Ceratosolen jucundus cplx 1
I JRAS03472_0151 Ceratosolen jucundus cplx 2
I JRAS03472_0155 Ceratosolen jucundus cplx 2
I JRAS03472_0156 Ceratosolen jucundus cplx 2
S JRAS03472_0101 Ceratosolen jucundus cplx 2
I JRAS03568_0253 Ceratosolen jucundus cplx 3
I JRAS03568_0251 Ceratosolen jucundus cplx 3
I JRAS03568_0252 Ceratosolen jucundus cplx 3
I JRAS01888_0151 Ceratosolen bisulcatus
I JRAS01888_0152 Ceratosolen bisulcatus
I JRAS01888_0153 Ceratosolen bisulcatus
S JRAS01888_0199 Ceratosolen bisulcatus
S JRAS01683_0195 Ceratosolen nsp046
S JRAS01683_0102 Ceratosolen nsp046
S JRAS01683_0101 Ceratosolen nsp046
I JRAS01683_0151 Ceratosolen nsp046
I JRAS01683_0155 Ceratosolen nsp046
I JRAS05988_0156 Ceratosolen nsp038
I JRAS05988_0155 Ceratosolen nsp038
S JRAS05988_0101 Ceratosolen nsp038

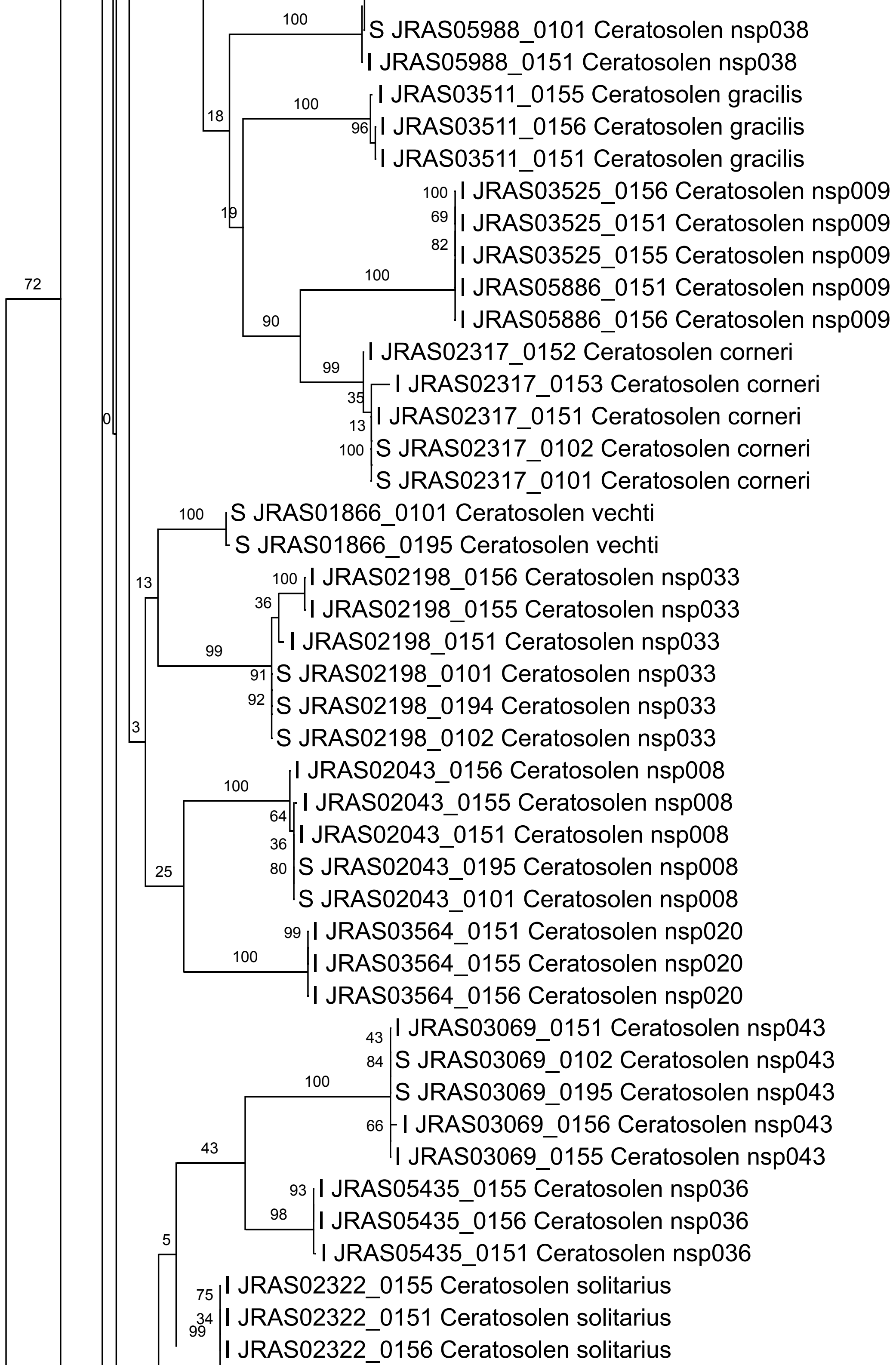
S JRAS05988_0101 Ceratosolen nsp038
I JRAS05988_0151 Ceratosolen nsp038
I JRAS03511_0155 Ceratosolen gracilis
I JRAS03511_0156 Ceratosolen gracilis
I JRAS03511_0151 Ceratosolen gracilis
I JRAS03525_0156 Ceratosolen nsp009
I JRAS03525_0151 Ceratosolen nsp009
I JRAS03525_0155 Ceratosolen nsp009
I JRAS05886_0151 Ceratosolen nsp009
I JRAS05886_0156 Ceratosolen nsp009
I JRAS02317_0152 Ceratosolen corneri
I JRAS02317_0153 Ceratosolen corneri
I JRAS02317_0151 Ceratosolen corneri
S JRAS02317_0102 Ceratosolen corneri
S JRAS02317_0101 Ceratosolen corneri
S JRAS01866_0101 Ceratosolen vechti
S JRAS01866_0195 Ceratosolen vechti
I JRAS02198_0156 Ceratosolen nsp033
I JRAS02198_0155 Ceratosolen nsp033
I JRAS02198_0151 Ceratosolen nsp033
S JRAS02198_0101 Ceratosolen nsp033
S JRAS02198_0194 Ceratosolen nsp033
S JRAS02198_0102 Ceratosolen nsp033
I JRAS02043_0156 Ceratosolen nsp008
I JRAS02043_0155 Ceratosolen nsp008
I JRAS02043_0151 Ceratosolen nsp008
S JRAS02043_0195 Ceratosolen nsp008
S JRAS02043_0101 Ceratosolen nsp008
I JRAS03564_0151 Ceratosolen nsp020
I JRAS03564_0155 Ceratosolen nsp020
I JRAS03564_0156 Ceratosolen nsp020
I JRAS03069_0151 Ceratosolen nsp043
S JRAS03069_0102 Ceratosolen nsp043
S JRAS03069_0195 Ceratosolen nsp043
I JRAS03069_0156 Ceratosolen nsp043
I JRAS03069_0155 Ceratosolen nsp043
I JRAS05435_0155 Ceratosolen nsp036
I JRAS05435_0156 Ceratosolen nsp036
I JRAS05435_0151 Ceratosolen nsp036
I JRAS02322_0155 Ceratosolen solitarius
I JRAS02322_0151 Ceratosolen solitarius
I JRAS02322_0156 Ceratosolen solitarius

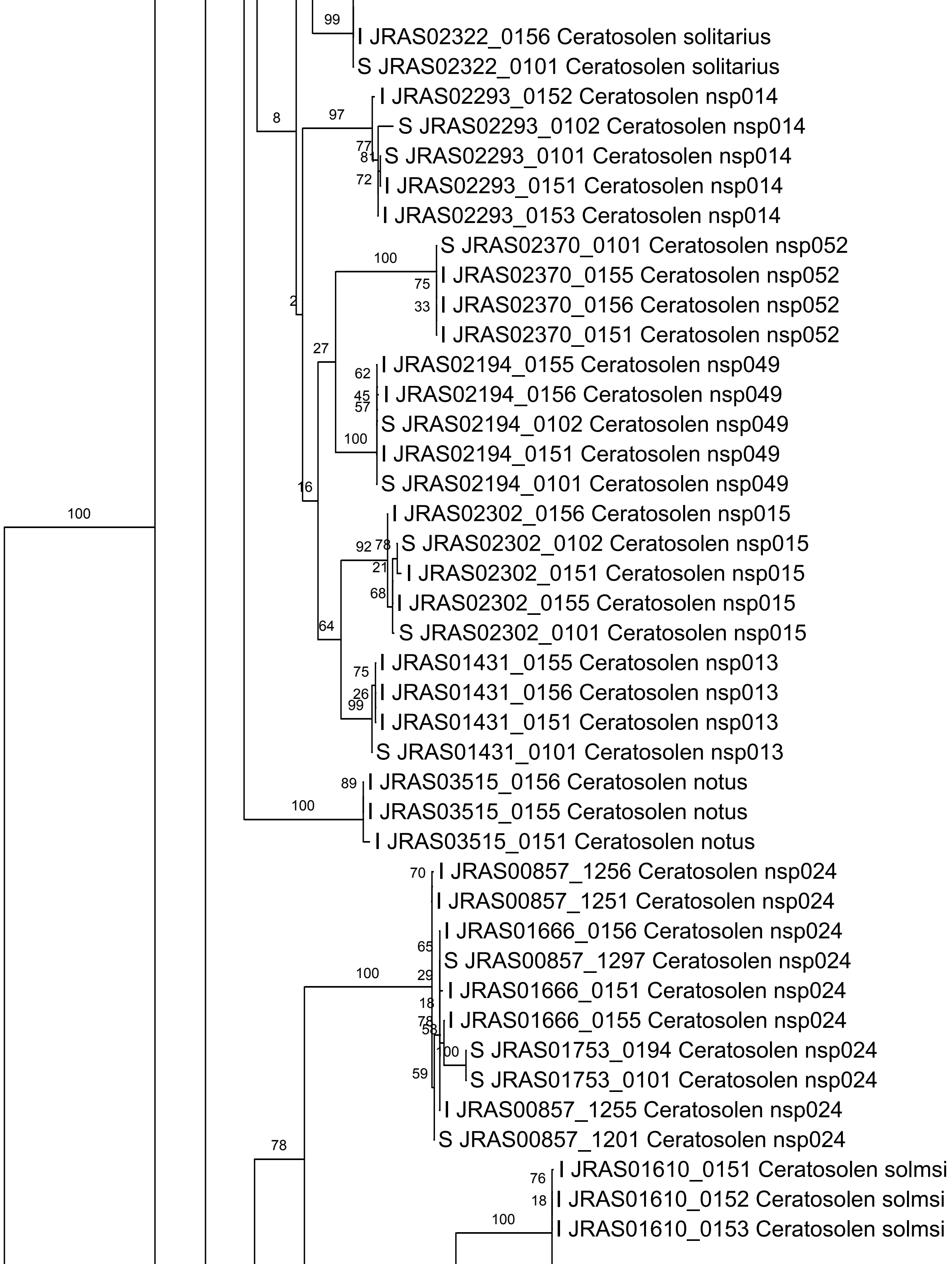

99
I JRAS02322_0156 Ceratosolen solitarius
S JRAS02322_0101 Ceratosolen solitarius
I JRAS02293_0152 Ceratosolen nsp014
8
97
S JRAS02293_0102 Ceratosolen nsp014
77
81
S JRAS02293_0101 Ceratosolen nsp014
72
I JRAS02293_0151 Ceratosolen nsp014
I JRAS02293_0153 Ceratosolen nsp014
S JRAS02370_0101 Ceratosolen nsp052
100
I JRAS02370_0155 Ceratosolen nsp052
75
I JRAS02370_0156 Ceratosolen nsp052
33
2
I JRAS02370_0151 Ceratosolen nsp052
27
I JRAS02194_0155 Ceratosolen nsp049
62
I JRAS02194_0156 Ceratosolen nsp049
45
57
S JRAS02194_0102 Ceratosolen nsp049
100
I JRAS02194_0151 Ceratosolen nsp049
S JRAS02194_0101 Ceratosolen nsp049
16
I JRAS02302_0156 Ceratosolen nsp015
100
92
78
S JRAS02302_0102 Ceratosolen nsp015
21
I JRAS02302_0151 Ceratosolen nsp015
68
I JRAS02302_0155 Ceratosolen nsp015
64
S JRAS02302_0101 Ceratosolen nsp015
I JRAS01431_0155 Ceratosolen nsp013
75
26
I JRAS01431_0156 Ceratosolen nsp013
99
I JRAS01431_0151 Ceratosolen nsp013
S JRAS01431_0101 Ceratosolen nsp013
89
I JRAS03515_0156 Ceratosolen notus
100
I JRAS03515_0155 Ceratosolen notus
I JRAS03515_0151 Ceratosolen notus
70
I JRAS00857_1256 Ceratosolen nsp024
I JRAS00857_1251 Ceratosolen nsp024
I JRAS01666_0156 Ceratosolen nsp024
65
S JRAS00857_1297 Ceratosolen nsp024
100
29
I JRAS01666_0151 Ceratosolen nsp024
18
I JRAS01666_0155 Ceratosolen nsp024
78
58
100
S JRAS01753_0194 Ceratosolen nsp024
59
S JRAS01753_0101 Ceratosolen nsp024
I JRAS00857_1255 Ceratosolen nsp024
S JRAS00857_1201 Ceratosolen nsp024
78
76
I JRAS01610_0151 Ceratosolen solmsi
18
I JRAS01610_0152 Ceratosolen solmsi
100
I JRAS01610_0153 Ceratosolen solmsi

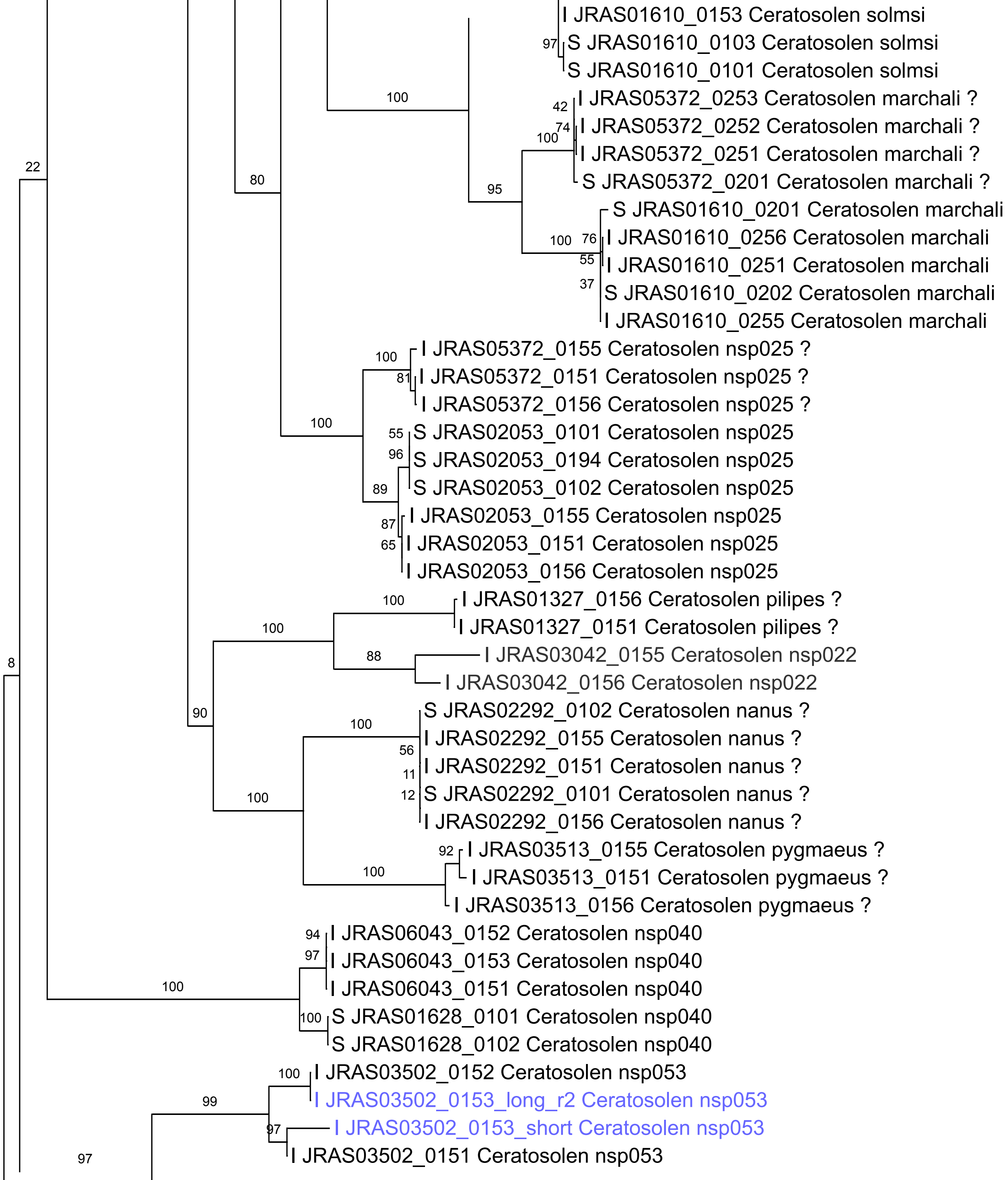
I JRAS01610_0153 Ceratosolen solmsi
S JRAS01610_0103 Ceratosolen solmsi
S JRAS01610_0101 Ceratosolen solmsi
I JRAS05372_0253 Ceratosolen marchali ?
I JRAS05372_0252 Ceratosolen marchali ?
I JRAS05372_0251 Ceratosolen marchali ?
S JRAS05372_0201 Ceratosolen marchali ?
S JRAS01610_0201 Ceratosolen marchali
I JRAS01610_0256 Ceratosolen marchali
I JRAS01610_0251 Ceratosolen marchali
S JRAS01610_0202 Ceratosolen marchali
I JRAS01610_0255 Ceratosolen marchali
I JRAS05372_0155 Ceratosolen nsp025 ?
I JRAS05372_0151 Ceratosolen nsp025 ?
I JRAS05372_0156 Ceratosolen nsp025 ?
S JRAS02053_0101 Ceratosolen nsp025
S JRAS02053_0194 Ceratosolen nsp025
S JRAS02053_0102 Ceratosolen nsp025
I JRAS02053_0155 Ceratosolen nsp025
I JRAS02053_0151 Ceratosolen nsp025
I JRAS02053_0156 Ceratosolen nsp025
I JRAS01327_0156 Ceratosolen pilipes ?
I JRAS01327_0151 Ceratosolen pilipes ?
I JRAS03042_0155 Ceratosolen nsp022
I JRAS03042_0156 Ceratosolen nsp022
S JRAS02292_0102 Ceratosolen nanus ?
I JRAS02292_0155 Ceratosolen nanus ?
I JRAS02292_0151 Ceratosolen nanus ?
S JRAS02292_0101 Ceratosolen nanus ?
I JRAS02292_0156 Ceratosolen nanus ?
I JRAS03513_0155 Ceratosolen pygmaeus ?
I JRAS03513_0151 Ceratosolen pygmaeus ?
I JRAS03513_0156 Ceratosolen pygmaeus ?
I JRAS06043_0152 Ceratosolen nsp040
I JRAS06043_0153 Ceratosolen nsp040
I JRAS06043_0151 Ceratosolen nsp040
S JRAS01628_0101 Ceratosolen nsp040
S JRAS01628_0102 Ceratosolen nsp040
I JRAS03502_0152 Ceratosolen nsp053
I JRAS03502_0153_long_r2 Ceratosolen nsp053
I JRAS03502_0153_short Ceratosolen nsp053
I JRAS03502_0151 Ceratosolen nsp053

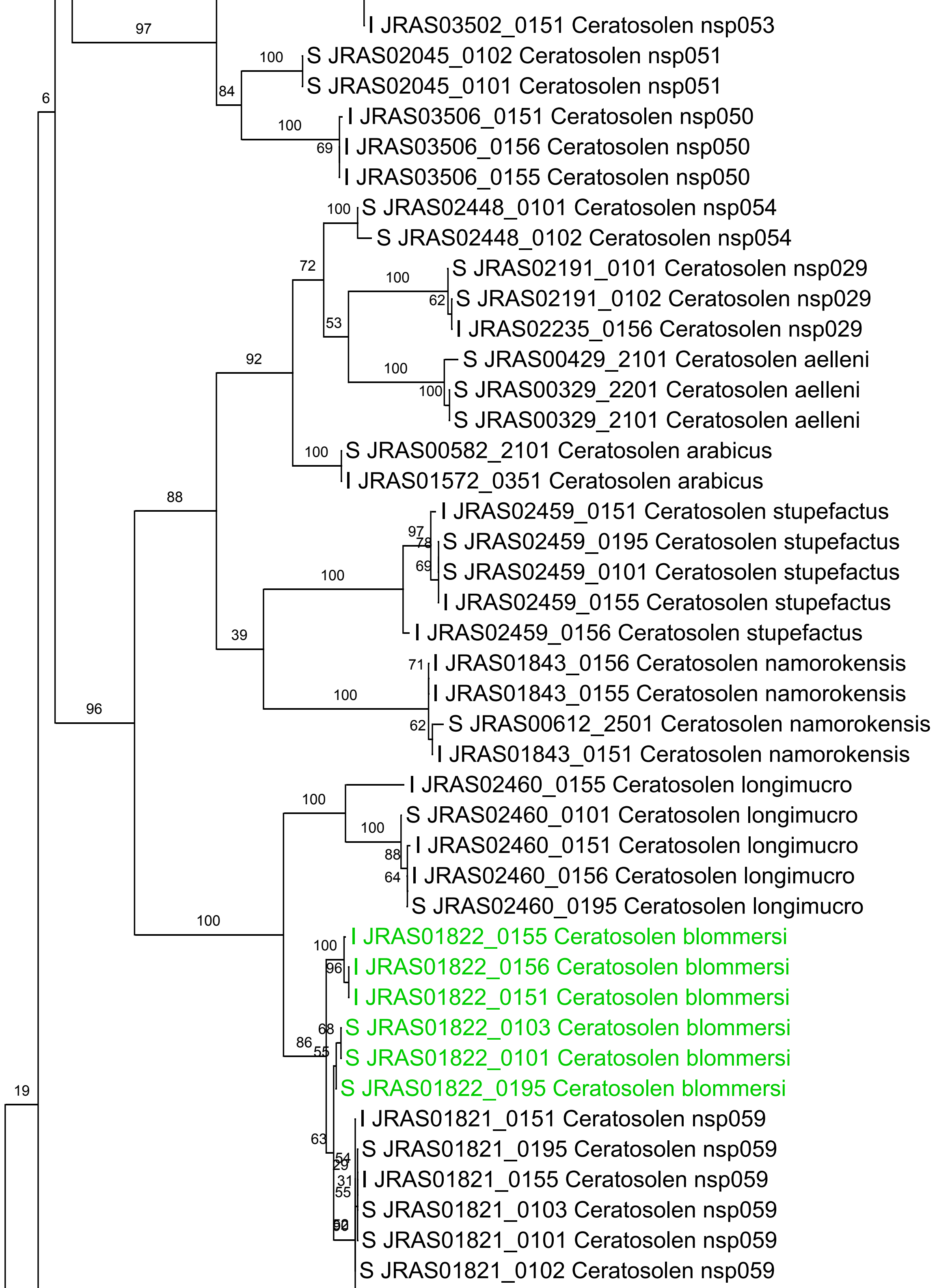
I JRAS03502_0151 Ceratosolen nsp053
S JRAS02045_0102 Ceratosolen nsp051
S JRAS02045_0101 Ceratosolen nsp051
I JRAS03506_0151 Ceratosolen nsp050
I JRAS03506_0156 Ceratosolen nsp050
I JRAS03506_0155 Ceratosolen nsp050
S JRAS02448_0101 Ceratosolen nsp054
S JRAS02448_0102 Ceratosolen nsp054
S JRAS02191_0101 Ceratosolen nsp029
S JRAS02191_0102 Ceratosolen nsp029
I JRAS02235_0156 Ceratosolen nsp029
S JRAS00429_2101 Ceratosolen aelleni
S JRAS00329_2201 Ceratosolen aelleni
S JRAS00329_2101 Ceratosolen aelleni
S JRAS00582_2101 Ceratosolen arabicus
I JRAS01572_0351 Ceratosolen arabicus
I JRAS02459_0151 Ceratosolen stupefactus
S JRAS02459_0195 Ceratosolen stupefactus
S JRAS02459_0101 Ceratosolen stupefactus
I JRAS02459_0155 Ceratosolen stupefactus
I JRAS02459_0156 Ceratosolen stupefactus
I JRAS01843_0156 Ceratosolen namorokensis
I JRAS01843_0155 Ceratosolen namorokensis
S JRAS00612_2501 Ceratosolen namorokensis
I JRAS01843_0151 Ceratosolen namorokensis
I JRAS02460_0155 Ceratosolen longimucro
S JRAS02460_0101 Ceratosolen longimucro
I JRAS02460_0151 Ceratosolen longimucro
I JRAS02460_0156 Ceratosolen longimucro
S JRAS02460_0195 Ceratosolen longimucro
I JRAS01822_0155 Ceratosolen blommersi
I JRAS01822_0156 Ceratosolen blommersi
I JRAS01822_0151 Ceratosolen blommersi
S JRAS01822_0103 Ceratosolen blommersi
S JRAS01822_0101 Ceratosolen blommersi
S JRAS01822_0195 Ceratosolen blommersi
I JRAS01821_0151 Ceratosolen nsp059
S JRAS01821_0195 Ceratosolen nsp059
I JRAS01821_0155 Ceratosolen nsp059
S JRAS01821_0103 Ceratosolen nsp059
S JRAS01821_0101 Ceratosolen nsp059
S JRAS01821_0102 Ceratosolen nsp059

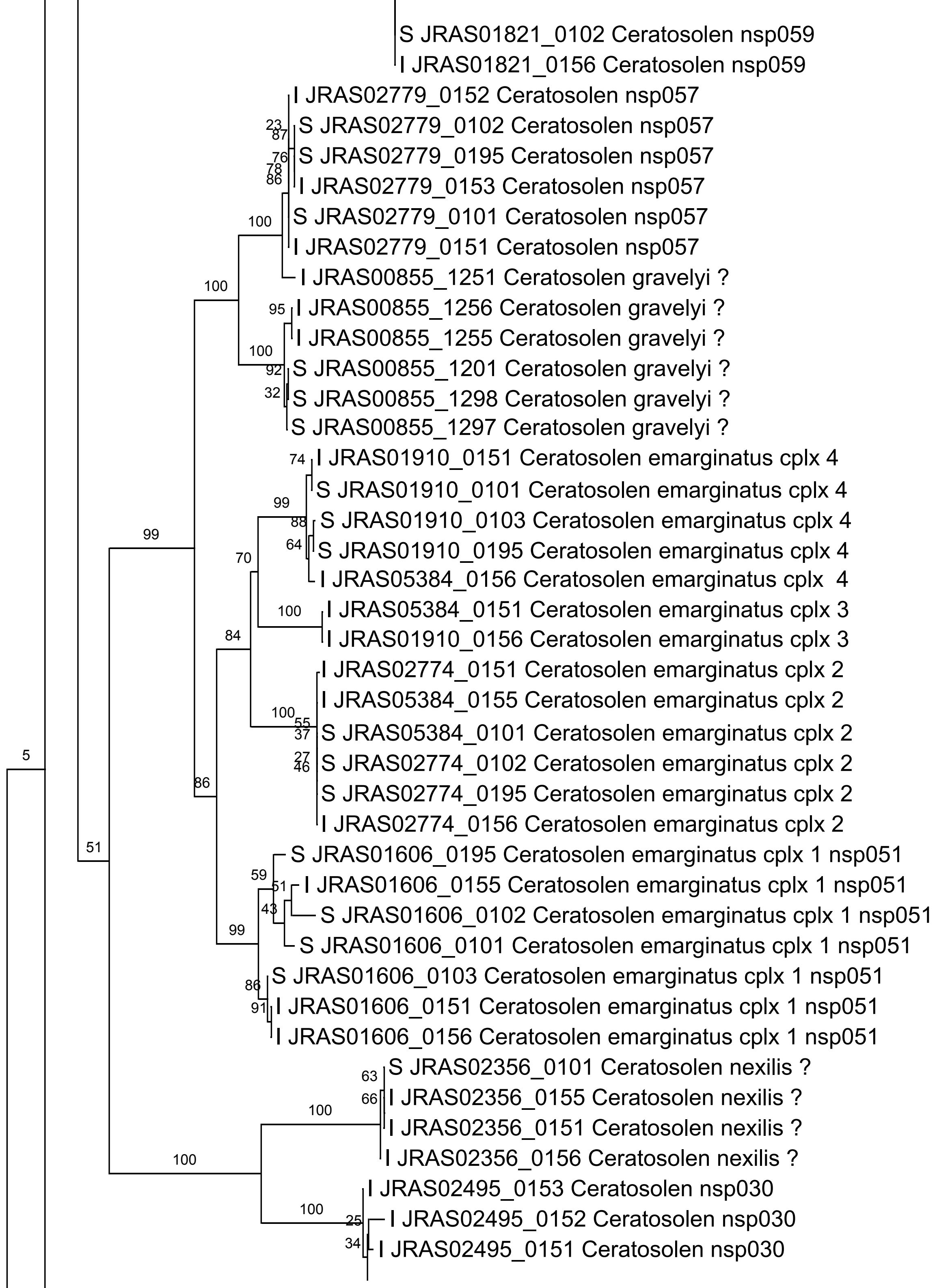
S JRAS01821_0102 Ceratosolen nsp059
I JRAS01821_0156 Ceratosolen nsp059
I JRAS02779_0152 Ceratosolen nsp057
S JRAS02779_0102 Ceratosolen nsp057
S JRAS02779_0195 Ceratosolen nsp057
I JRAS02779_0153 Ceratosolen nsp057
S JRAS02779_0101 Ceratosolen nsp057
I JRAS02779_0151 Ceratosolen nsp057
I JRAS00855_1251 Ceratosolen gravelyi ?
I JRAS00855_1256 Ceratosolen gravelyi ?
I JRAS00855_1255 Ceratosolen gravelyi ?
S JRAS00855_1201 Ceratosolen gravelyi ?
S JRAS00855_1298 Ceratosolen gravelyi ?
S JRAS00855_1297 Ceratosolen gravelyi ?
I JRAS01910_0151 Ceratosolen emarginatus cplx 4
S JRAS01910_0101 Ceratosolen emarginatus cplx 4
S JRAS01910_0103 Ceratosolen emarginatus cplx 4
S JRAS01910_0195 Ceratosolen emarginatus cplx 4
I JRAS05384_0156 Ceratosolen emarginatus cplx 4
I JRAS05384_0151 Ceratosolen emarginatus cplx 3
I JRAS01910_0156 Ceratosolen emarginatus cplx 3
I JRAS02774_0151 Ceratosolen emarginatus cplx 2
I JRAS05384_0155 Ceratosolen emarginatus cplx 2
S JRAS05384_0101 Ceratosolen emarginatus cplx 2
S JRAS02774_0102 Ceratosolen emarginatus cplx 2
S JRAS02774_0195 Ceratosolen emarginatus cplx 2
I JRAS02774_0156 Ceratosolen emarginatus cplx 2
S JRAS01606_0195 Ceratosolen emarginatus cplx 1 nsp051
I JRAS01606_0155 Ceratosolen emarginatus cplx 1 nsp051
S JRAS01606_0102 Ceratosolen emarginatus cplx 1 nsp051
S JRAS01606_0101 Ceratosolen emarginatus cplx 1 nsp051
S JRAS01606_0103 Ceratosolen emarginatus cplx 1 nsp051
I JRAS01606_0151 Ceratosolen emarginatus cplx 1 nsp051
I JRAS01606_0156 Ceratosolen emarginatus cplx 1 nsp051
S JRAS02356_0101 Ceratosolen nexilis ?
I JRAS02356_0155 Ceratosolen nexilis ?
I JRAS02356_0151 Ceratosolen nexilis ?
I JRAS02356_0156 Ceratosolen nexilis ?
I JRAS02495_0153 Ceratosolen nsp030
I JRAS02495_0152 Ceratosolen nsp030
I JRAS02495_0151 Ceratosolen nsp030

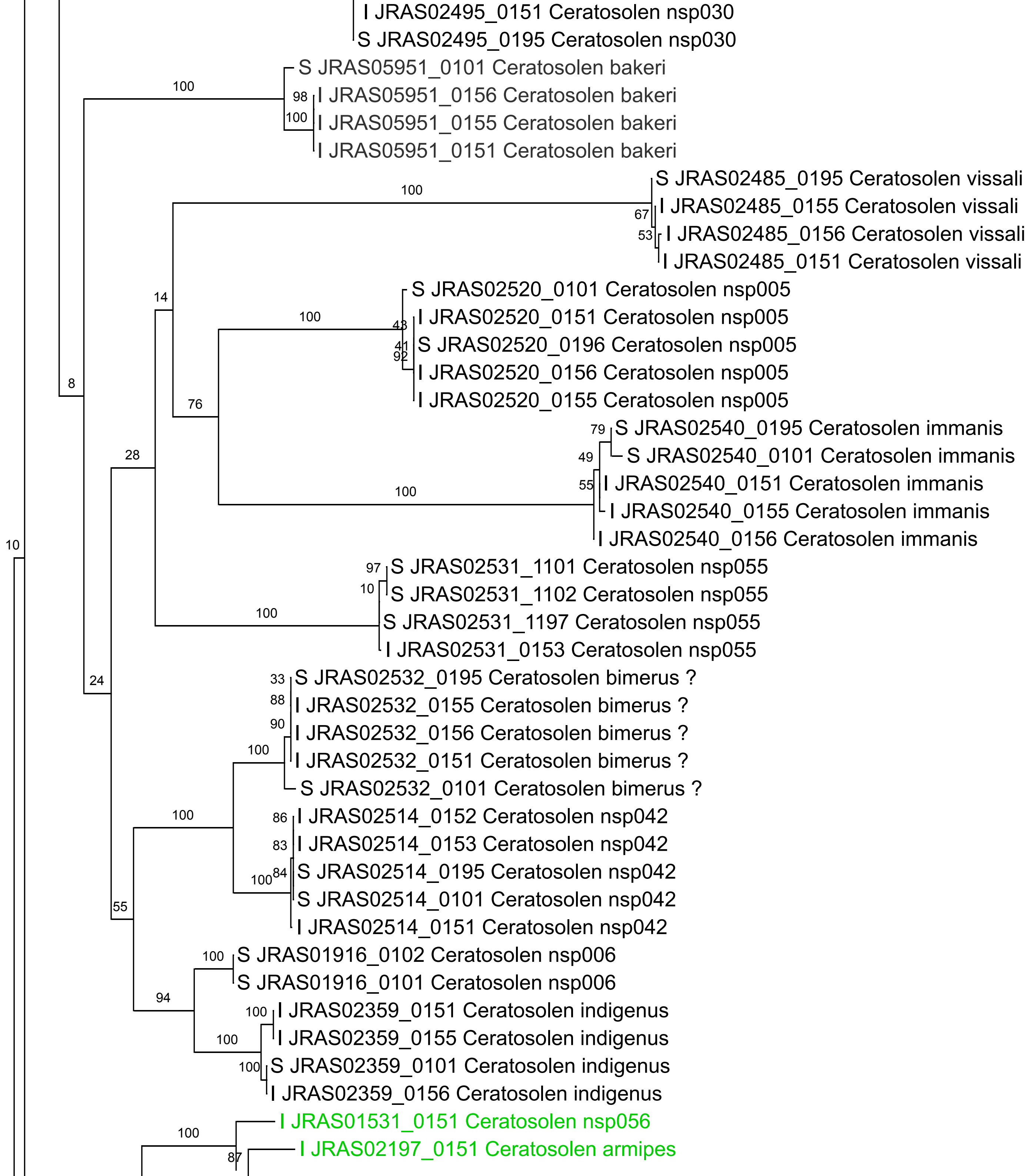
I JRAS02495_0151 Ceratosolen nsp030
S JRAS02495_0195 Ceratosolen nsp030
S JRAS05951_0101 Ceratosolen bakeri
I JRAS05951_0156 Ceratosolen bakeri
I JRAS05951_0155 Ceratosolen bakeri
I JRAS05951_0151 Ceratosolen bakeri
S JRAS02485_0195 Ceratosolen vissali
I JRAS02485_0155 Ceratosolen vissali
I JRAS02485_0156 Ceratosolen vissali
I JRAS02485_0151 Ceratosolen vissali
S JRAS02520_0101 Ceratosolen nsp005
I JRAS02520_0151 Ceratosolen nsp005
S JRAS02520_0196 Ceratosolen nsp005
I JRAS02520_0156 Ceratosolen nsp005
I JRAS02520_0155 Ceratosolen nsp005
S JRAS02540_0195 Ceratosolen immanis
S JRAS02540_0101 Ceratosolen immanis
I JRAS02540_0151 Ceratosolen immanis
I JRAS02540_0155 Ceratosolen immanis
I JRAS02540_0156 Ceratosolen immanis
S JRAS02531_1101 Ceratosolen nsp055
S JRAS02531_1102 Ceratosolen nsp055
S JRAS02531_1197 Ceratosolen nsp055
I JRAS02531_0153 Ceratosolen nsp055
S JRAS02532_0195 Ceratosolen bimerus ?
I JRAS02532_0155 Ceratosolen bimerus ?
I JRAS02532_0156 Ceratosolen bimerus ?
I JRAS02532_0151 Ceratosolen bimerus ?
S JRAS02532_0101 Ceratosolen bimerus ?
I JRAS02514_0152 Ceratosolen nsp042
I JRAS02514_0153 Ceratosolen nsp042
S JRAS02514_0195 Ceratosolen nsp042
S JRAS02514_0101 Ceratosolen nsp042
I JRAS02514_0151 Ceratosolen nsp042
S JRAS01916_0102 Ceratosolen nsp006
S JRAS01916_0101 Ceratosolen nsp006
I JRAS02359_0151 Ceratosolen indigenus
I JRAS02359_0155 Ceratosolen indigenus
S JRAS02359_0101 Ceratosolen indigenus
I JRAS02359_0156 Ceratosolen indigenus
I JRAS01531_0151 Ceratosolen nsp056
I JRAS02197_0151 Ceratosolen armipes

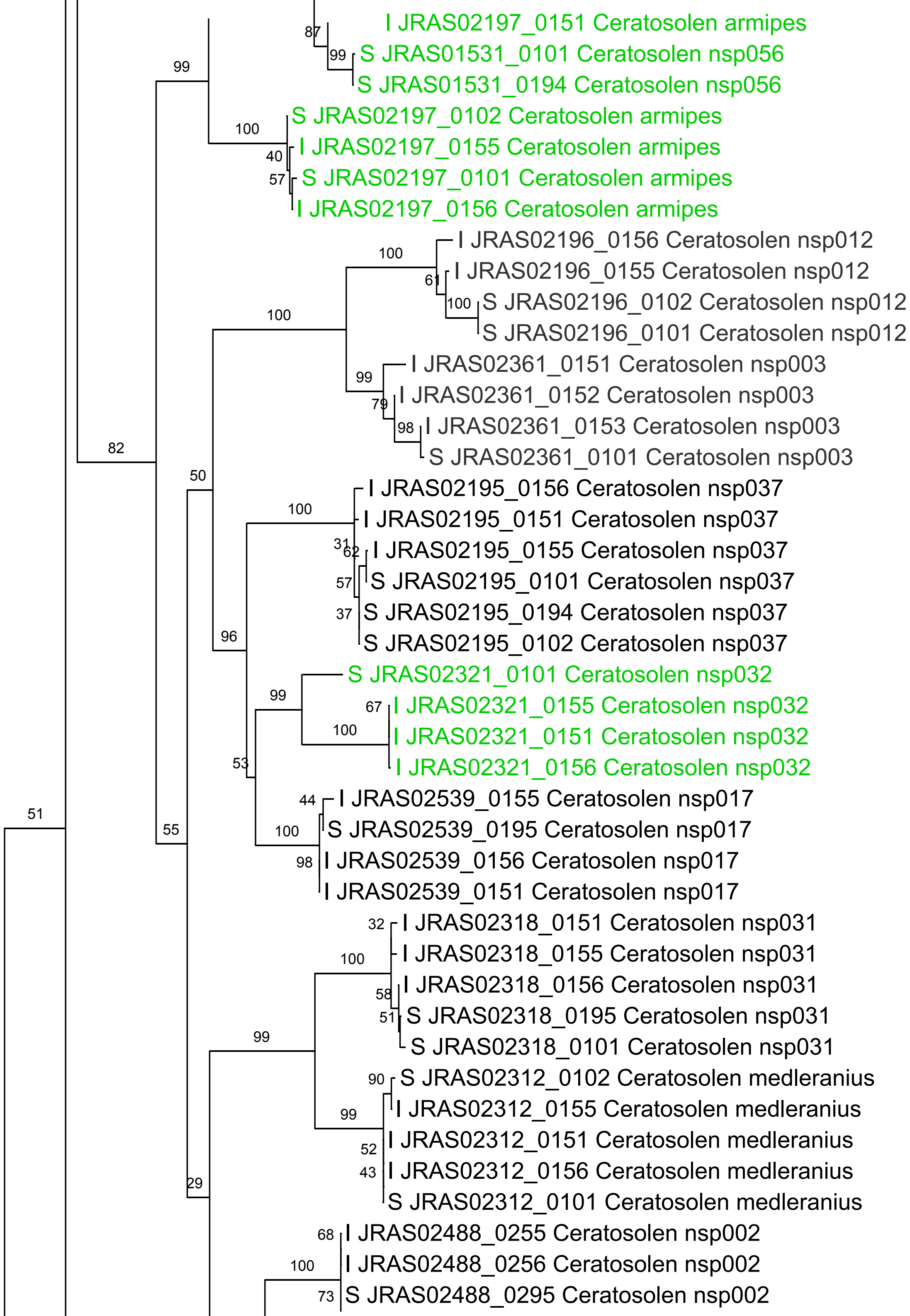
I JRAS02197_0151 Ceratosolen armipes
S JRAS01531_0101 Ceratosolen nsp056
S JRAS01531_0194 Ceratosolen nsp056
S JRAS02197_0102 Ceratosolen armipes
I JRAS02197_0155 Ceratosolen armipes
S JRAS02197_0101 Ceratosolen armipes
I JRAS02197_0156 Ceratosolen armipes
I JRAS02196_0156 Ceratosolen nsp012
I JRAS02196_0155 Ceratosolen nsp012
S JRAS02196_0102 Ceratosolen nsp012
S JRAS02196_0101 Ceratosolen nsp012
I JRAS02361_0151 Ceratosolen nsp003
I JRAS02361_0152 Ceratosolen nsp003
I JRAS02361_0153 Ceratosolen nsp003
S JRAS02361_0101 Ceratosolen nsp003
I JRAS02195_0156 Ceratosolen nsp037
I JRAS02195_0151 Ceratosolen nsp037
I JRAS02195_0155 Ceratosolen nsp037
S JRAS02195_0101 Ceratosolen nsp037
S JRAS02195_0194 Ceratosolen nsp037
S JRAS02195_0102 Ceratosolen nsp037
S JRAS02321_0101 Ceratosolen nsp032
I JRAS02321_0155 Ceratosolen nsp032
I JRAS02321_0151 Ceratosolen nsp032
I JRAS02321_0156 Ceratosolen nsp032
I JRAS02539_0155 Ceratosolen nsp017
S JRAS02539_0195 Ceratosolen nsp017
I JRAS02539_0156 Ceratosolen nsp017
I JRAS02539_0151 Ceratosolen nsp017
I JRAS02318_0151 Ceratosolen nsp031
I JRAS02318_0155 Ceratosolen nsp031
I JRAS02318_0156 Ceratosolen nsp031
S JRAS02318_0195 Ceratosolen nsp031
S JRAS02318_0101 Ceratosolen nsp031
S JRAS02312_0102 Ceratosolen medleranius
I JRAS02312_0155 Ceratosolen medleranius
I JRAS02312_0151 Ceratosolen medleranius
I JRAS02312_0156 Ceratosolen medleranius
S JRAS02312_0101 Ceratosolen medleranius
I JRAS02488_0255 Ceratosolen nsp002
I JRAS02488_0256 Ceratosolen nsp002
S JRAS02488_0295 Ceratosolen nsp002

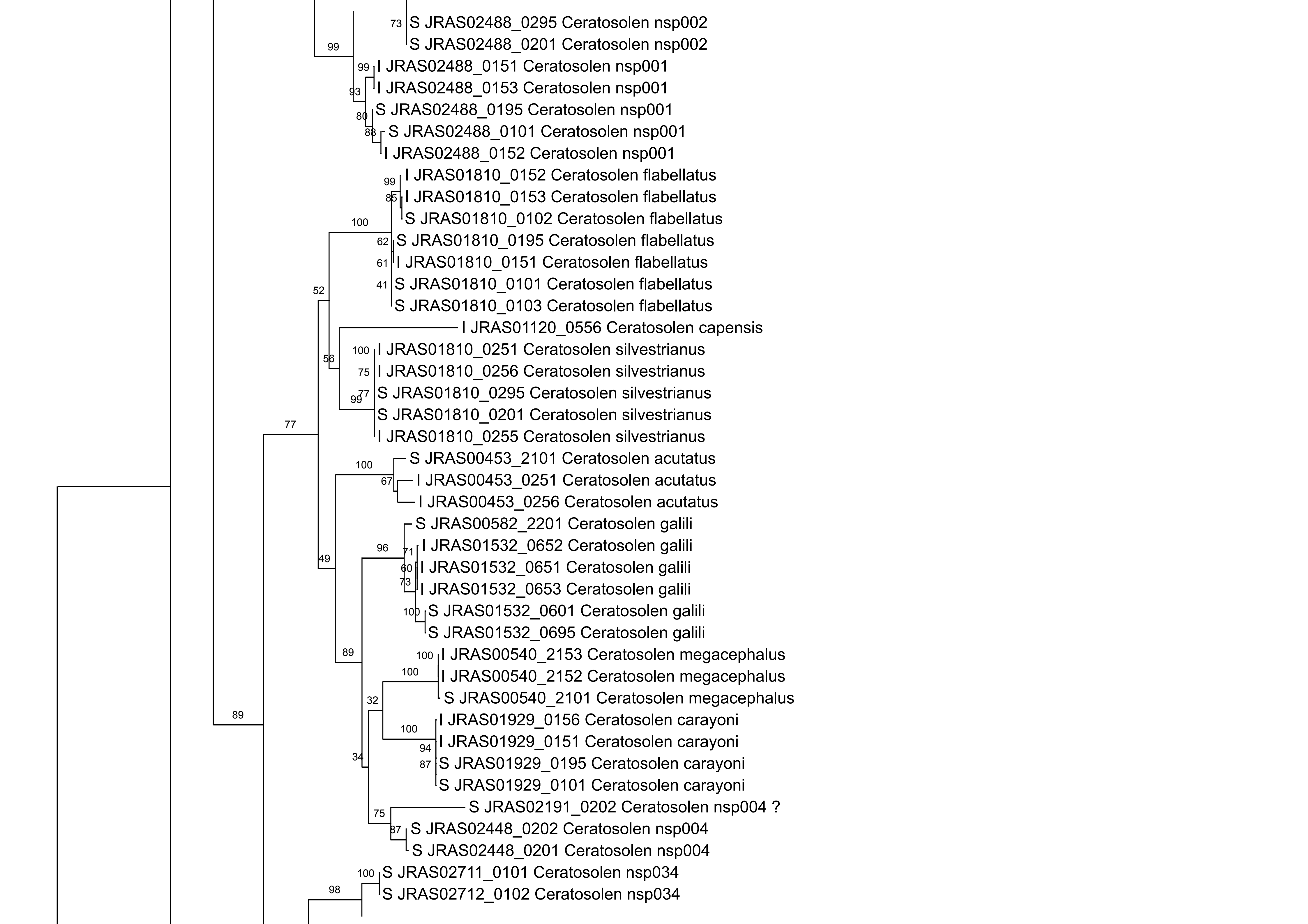
S JRAS02488_0295 Ceratosolen nsp002
S JRAS02488_0201 Ceratosolen nsp002
I JRAS02488_0151 Ceratosolen nsp001
I JRAS02488_0153 Ceratosolen nsp001
S JRAS02488_0195 Ceratosolen nsp001
S JRAS02488_0101 Ceratosolen nsp001
I JRAS02488_0152 Ceratosolen nsp001
I JRAS01810_0152 Ceratosolen flabellatus
I JRAS01810_0153 Ceratosolen flabellatus
S JRAS01810_0102 Ceratosolen flabellatus
S JRAS01810_0195 Ceratosolen flabellatus
I JRAS01810_0151 Ceratosolen flabellatus
S JRAS01810_0101 Ceratosolen flabellatus
S JRAS01810_0103 Ceratosolen flabellatus
I JRAS01120_0556 Ceratosolen capensis
I JRAS01810_0251 Ceratosolen silvestrianus
I JRAS01810_0256 Ceratosolen silvestrianus
S JRAS01810_0295 Ceratosolen silvestrianus
S JRAS01810_0201 Ceratosolen silvestrianus
I JRAS01810_0255 Ceratosolen silvestrianus
S JRAS00453_2101 Ceratosolen acutatus
I JRAS00453_0251 Ceratosolen acutatus
I JRAS00453_0256 Ceratosolen acutatus
S JRAS00582_2201 Ceratosolen galili
I JRAS01532_0652 Ceratosolen galili
I JRAS01532_0651 Ceratosolen galili
I JRAS01532_0653 Ceratosolen galili
S JRAS01532_0601 Ceratosolen galili
S JRAS01532_0695 Ceratosolen galili
I JRAS00540_2153 Ceratosolen megacephalus
I JRAS00540_2152 Ceratosolen megacephalus
S JRAS00540_2101 Ceratosolen megacephalus
I JRAS01929_0156 Ceratosolen carayoni
I JRAS01929_0151 Ceratosolen carayoni
S JRAS01929_0195 Ceratosolen carayoni
S JRAS01929_0101 Ceratosolen carayoni
S JRAS02191_0202 Ceratosolen nsp004 ?
S JRAS02448_0202 Ceratosolen nsp004
S JRAS02448_0201 Ceratosolen nsp004
S JRAS02711_0101 Ceratosolen nsp034
S JRAS02712_0102 Ceratosolen nsp034

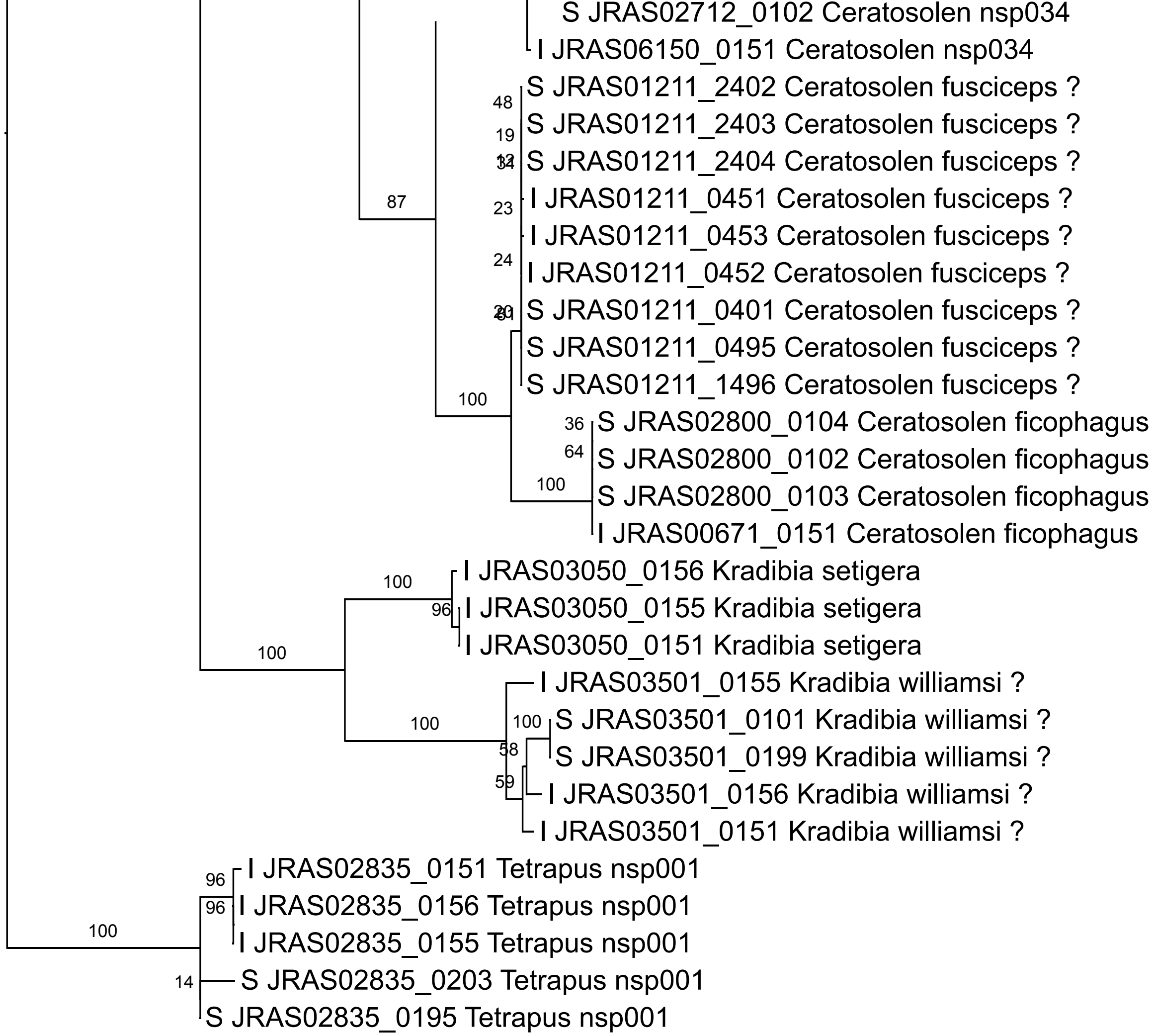

S JRAS02712_0102 Ceratosolen nsp034
I JRAS06150_0151 Ceratosolen nsp034
S JRAS01211_2402 Ceratosolen fusciceps ?
S JRAS01211_2403 Ceratosolen fusciceps ?
S JRAS01211_2404 Ceratosolen fusciceps ?
I JRAS01211_0451 Ceratosolen fusciceps ?
I JRAS01211_0453 Ceratosolen fusciceps ?
I JRAS01211_0452 Ceratosolen fusciceps ?
S JRAS01211_0401 Ceratosolen fusciceps ?
S JRAS01211_0495 Ceratosolen fusciceps ?
S JRAS01211_1496 Ceratosolen fusciceps ?
S JRAS02800_0104 Ceratosolen ficophagus
S JRAS02800_0102 Ceratosolen ficophagus
S JRAS02800_0103 Ceratosolen ficophagus
I JRAS00671_0151 Ceratosolen ficophagus
I JRAS03050_0156 Kradibia setigera
I JRAS03050_0155 Kradibia setigera
I JRAS03050_0151 Kradibia setigera
I JRAS03501_0155 Kradibia williamsi ?
S JRAS03501_0101 Kradibia williamsi ?
S JRAS03501_0199 Kradibia williamsi ?
I JRAS03501_0156 Kradibia williamsi ?
I JRAS03501_0151 Kradibia williamsi ?
I JRAS02835_0151 Tetrapus nsp001
I JRAS02835_0156 Tetrapus nsp001
I JRAS02835_0155 Tetrapus nsp001
S JRAS02835_0203 Tetrapus nsp001
S JRAS02835_0195 Tetrapus nsp001
48
19
23
24
87
100
36
64
100
100
96
100
100
100
58
59
96
96
100
14

Fig S2. RAxML tree for the Cytb data set (Miseq +Sanger) (BP : 1000 replicates).

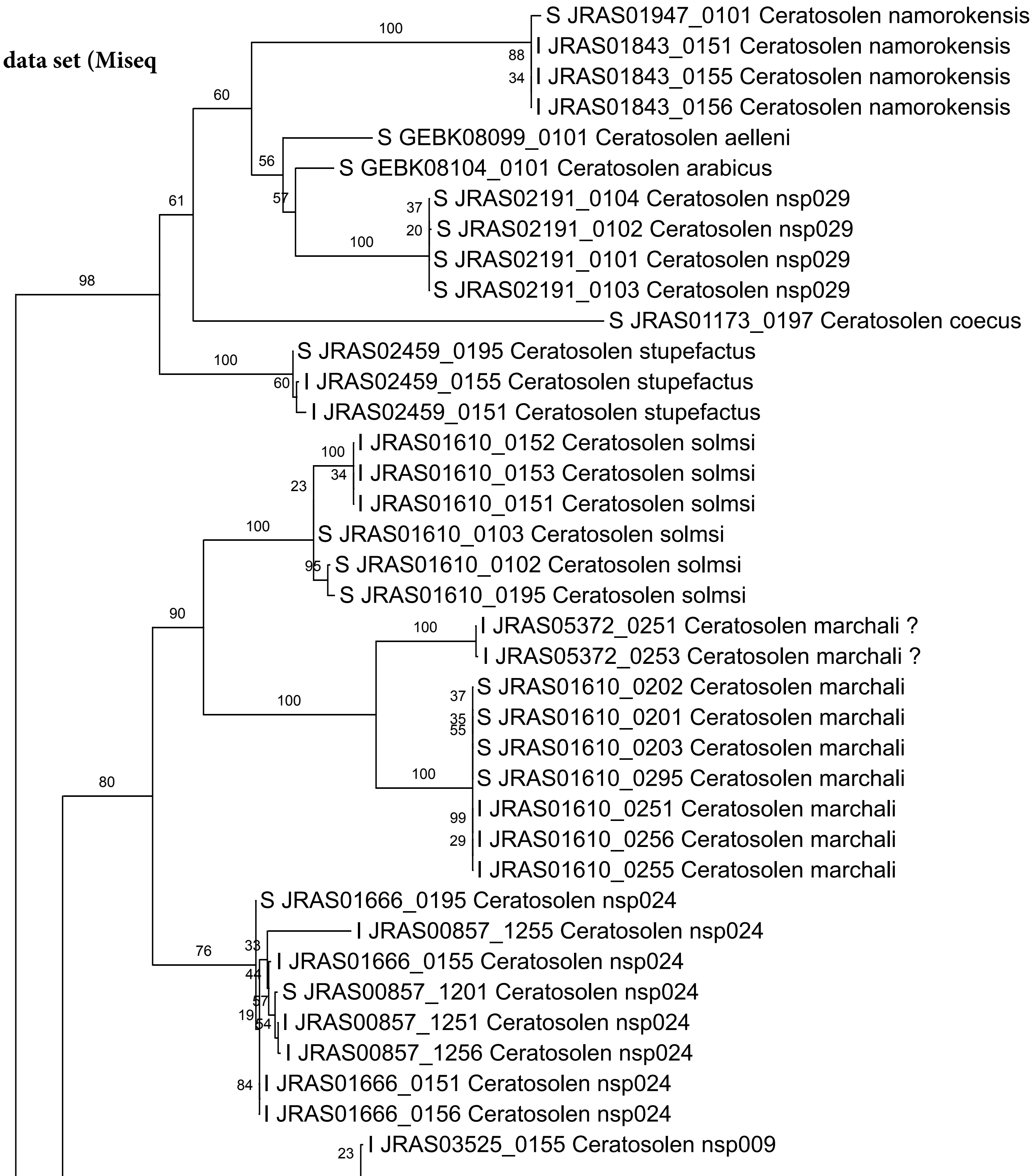

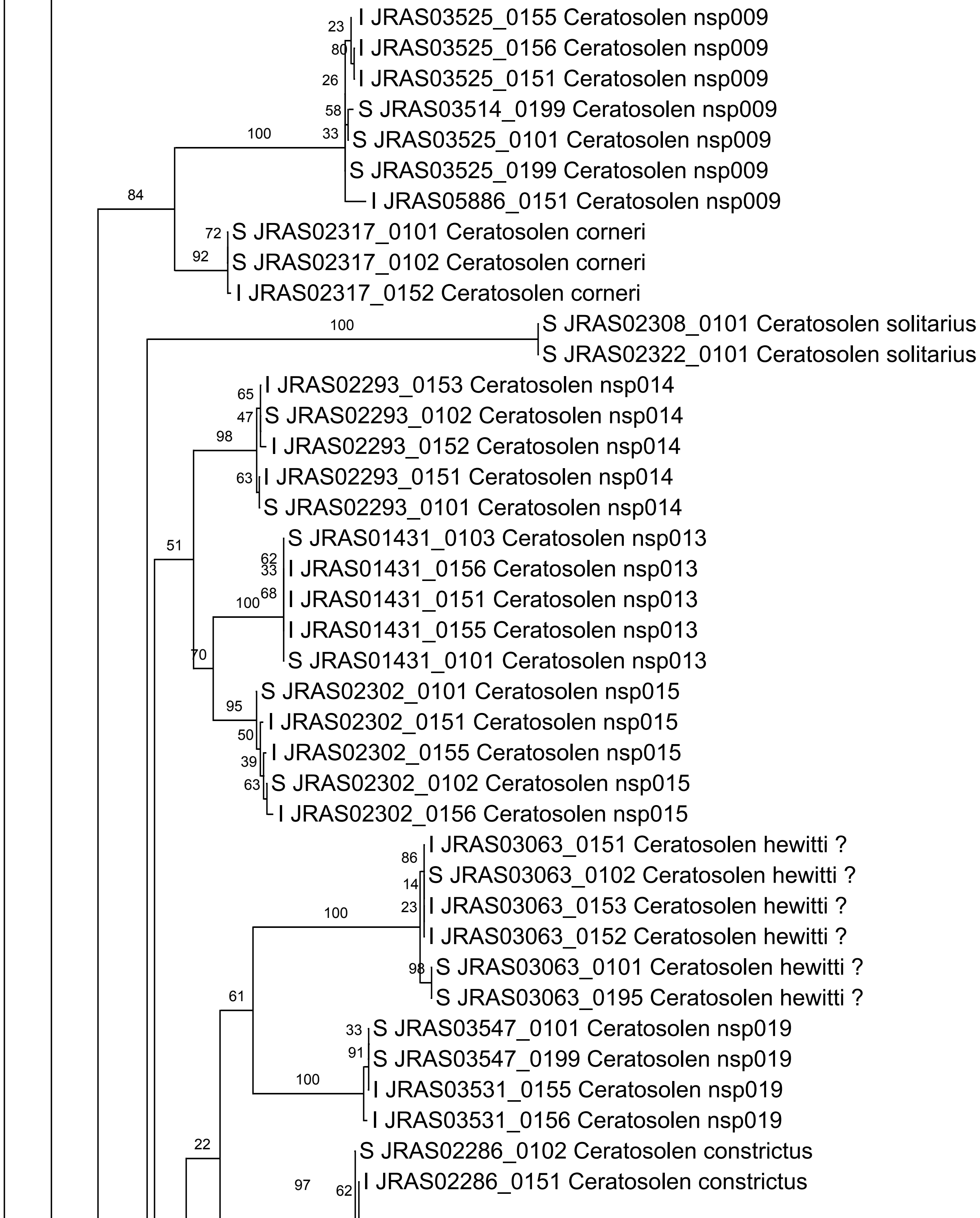
I JRAS03525_0155 Ceratosolen nsp009
I JRAS03525_0156 Ceratosolen nsp009
I JRAS03525_0151 Ceratosolen nsp009
S JRAS03514_0199 Ceratosolen nsp009
S JRAS03525_0101 Ceratosolen nsp009
S JRAS03525_0199 Ceratosolen nsp009
I JRAS05886_0151 Ceratosolen nsp009
S JRAS02317_0101 Ceratosolen corneri
S JRAS02317_0102 Ceratosolen corneri
I JRAS02317_0152 Ceratosolen corneri
S JRAS02308_0101 Ceratosolen solitarius
S JRAS02322_0101 Ceratosolen solitarius
I JRAS02293_0153 Ceratosolen nsp014
S JRAS02293_0102 Ceratosolen nsp014
I JRAS02293_0152 Ceratosolen nsp014
I JRAS02293_0151 Ceratosolen nsp014
S JRAS02293_0101 Ceratosolen nsp014
S JRAS01431_0103 Ceratosolen nsp013
I JRAS01431_0156 Ceratosolen nsp013
I JRAS01431_0151 Ceratosolen nsp013
I JRAS01431_0155 Ceratosolen nsp013
S JRAS01431_0101 Ceratosolen nsp013
S JRAS02302_0101 Ceratosolen nsp015
I JRAS02302_0151 Ceratosolen nsp015
I JRAS02302_0155 Ceratosolen nsp015
S JRAS02302_0102 Ceratosolen nsp015
I JRAS02302_0156 Ceratosolen nsp015
I JRAS03063_0151 Ceratosolen hewitti ?
S JRAS03063_0102 Ceratosolen hewitti ?
I JRAS03063_0153 Ceratosolen hewitti ?
I JRAS03063_0152 Ceratosolen hewitti ?
S JRAS03063_0101 Ceratosolen hewitti ?
S JRAS03063_0195 Ceratosolen hewitti ?
S JRAS03547_0101 Ceratosolen nsp019
S JRAS03547_0199 Ceratosolen nsp019
I JRAS03531_0155 Ceratosolen nsp019
I JRAS03531_0156 Ceratosolen nsp019
S JRAS02286_0102 Ceratosolen constrictus
I JRAS02286_0151 Ceratosolen constrictus

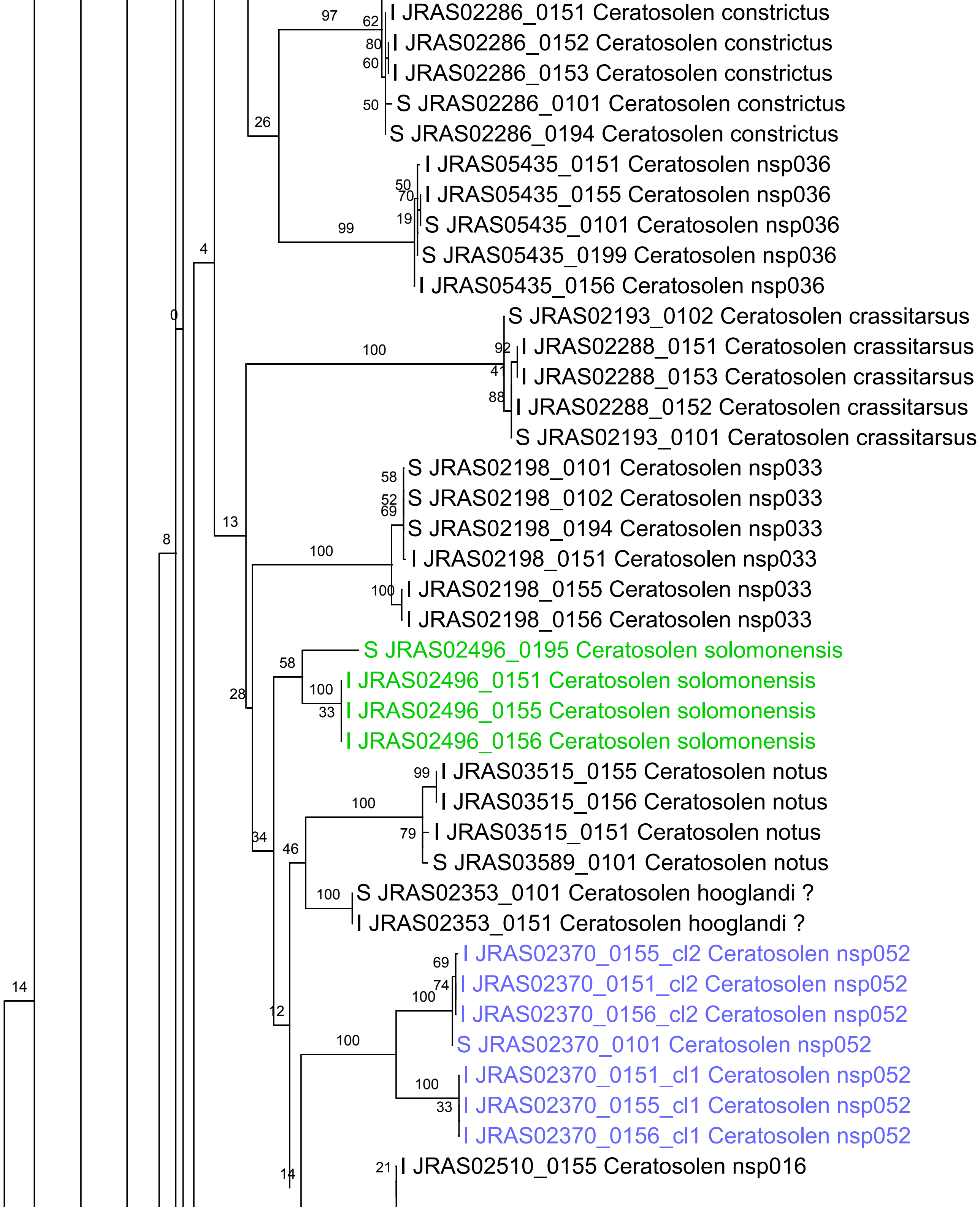
I JRAS02286_0151 Ceratosolen constrictus
I JRAS02286_0152 Ceratosolen constrictus
I JRAS02286_0153 Ceratosolen constrictus
S JRAS02286_0101 Ceratosolen constrictus
S JRAS02286_0194 Ceratosolen constrictus
I JRAS05435_0151 Ceratosolen nsp036
I JRAS05435_0155 Ceratosolen nsp036
S JRAS05435_0101 Ceratosolen nsp036
S JRAS05435_0199 Ceratosolen nsp036
I JRAS05435_0156 Ceratosolen nsp036
S JRAS02193_0102 Ceratosolen crassitarsus
I JRAS02288_0151 Ceratosolen crassitarsus
I JRAS02288_0153 Ceratosolen crassitarsus
I JRAS02288_0152 Ceratosolen crassitarsus
S JRAS02193_0101 Ceratosolen crassitarsus
S JRAS02198_0101 Ceratosolen nsp033
S JRAS02198_0102 Ceratosolen nsp033
S JRAS02198_0194 Ceratosolen nsp033
I JRAS02198_0151 Ceratosolen nsp033
I JRAS02198_0155 Ceratosolen nsp033
I JRAS02198_0156 Ceratosolen nsp033
S JRAS02496_0195 Ceratosolen solomonensis
I JRAS02496_0151 Ceratosolen solomonensis
I JRAS02496_0155 Ceratosolen solomonensis
I JRAS02496_0156 Ceratosolen solomonensis
I JRAS03515_0155 Ceratosolen notus
I JRAS03515_0156 Ceratosolen notus
I JRAS03515_0151 Ceratosolen notus
S JRAS03589_0101 Ceratosolen notus
S JRAS02353_0101 Ceratosolen hooglandi ?
I JRAS02353_0151 Ceratosolen hooglandi ?
I JRAS02370_0155_cl2 Ceratosolen nsp052
I JRAS02370_0151_cl2 Ceratosolen nsp052
I JRAS02370_0156_cl2 Ceratosolen nsp052
S JRAS02370_0101 Ceratosolen nsp052
I JRAS02370_0151_cl1 Ceratosolen nsp052
I JRAS02370_0155_cl1 Ceratosolen nsp052
I JRAS02370_0156_cl1 Ceratosolen nsp052
I JRAS02510_0155 Ceratosolen nsp016

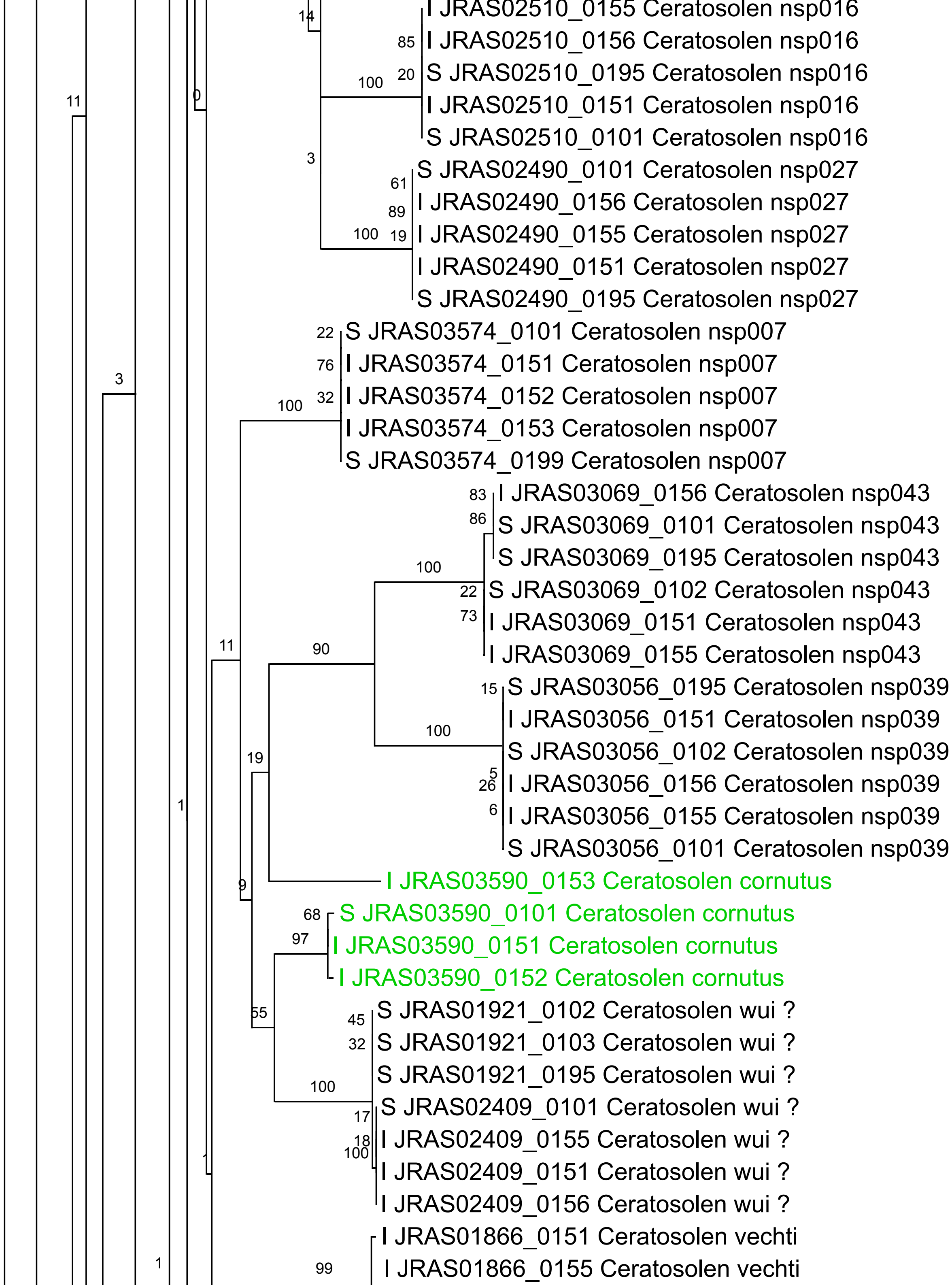
I JRAS02510_0155 Ceratosolen nsp016
I JRAS02510_0156 Ceratosolen nsp016
S JRAS02510_0195 Ceratosolen nsp016
I JRAS02510_0151 Ceratosolen nsp016
S JRAS02510_0101 Ceratosolen nsp016
S JRAS02490_0101 Ceratosolen nsp027
I JRAS02490_0156 Ceratosolen nsp027
I JRAS02490_0155 Ceratosolen nsp027
I JRAS02490_0151 Ceratosolen nsp027
S JRAS02490_0195 Ceratosolen nsp027
S JRAS03574_0101 Ceratosolen nsp007
I JRAS03574_0151 Ceratosolen nsp007
I JRAS03574_0152 Ceratosolen nsp007
I JRAS03574_0153 Ceratosolen nsp007
S JRAS03574_0199 Ceratosolen nsp007
I JRAS03069_0156 Ceratosolen nsp043
S JRAS03069_0101 Ceratosolen nsp043
S JRAS03069_0195 Ceratosolen nsp043
S JRAS03069_0102 Ceratosolen nsp043
I JRAS03069_0151 Ceratosolen nsp043
I JRAS03069_0155 Ceratosolen nsp043
S JRAS03056_0195 Ceratosolen nsp039
I JRAS03056_0151 Ceratosolen nsp039
S JRAS03056_0102 Ceratosolen nsp039
I JRAS03056_0156 Ceratosolen nsp039
I JRAS03056_0155 Ceratosolen nsp039
S JRAS03056_0101 Ceratosolen nsp039
I JRAS03590_0153 Ceratosolen cornutus
S JRAS03590_0101 Ceratosolen cornutus
I JRAS03590_0151 Ceratosolen cornutus
I JRAS03590_0152 Ceratosolen cornutus
S JRAS01921_0102 Ceratosolen wui ?
S JRAS01921_0103 Ceratosolen wui ?
S JRAS01921_0195 Ceratosolen wui ?
S JRAS02409_0101 Ceratosolen wui ?
I JRAS02409_0155 Ceratosolen wui ?
I JRAS02409_0151 Ceratosolen wui ?
I JRAS02409_0156 Ceratosolen wui ?
I JRAS01866_0151 Ceratosolen vechti
I JRAS01866_0155 Ceratosolen vechti

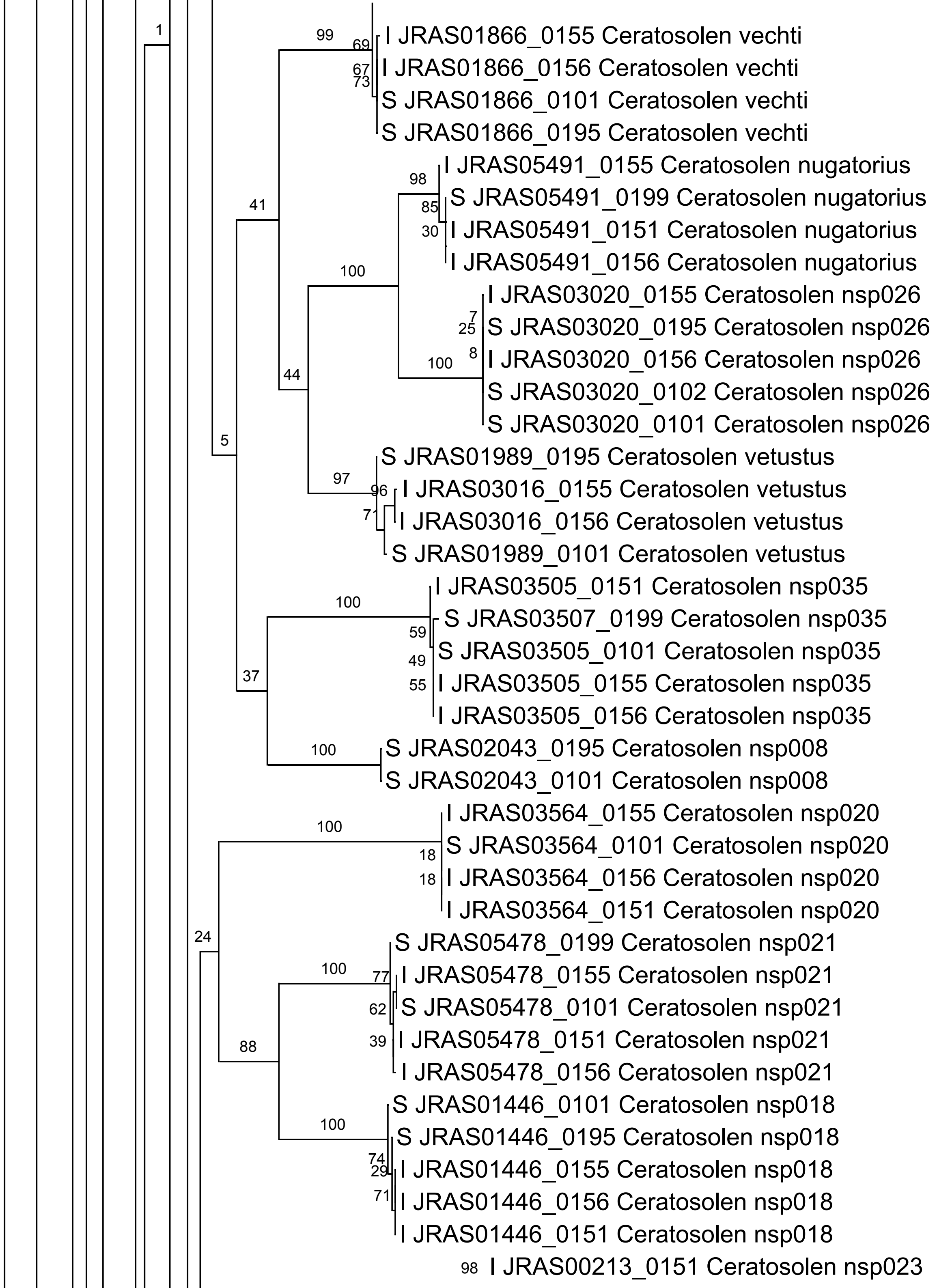
I JRAS01866_0155 Ceratosolen vechti
I JRAS01866_0156 Ceratosolen vechti
S JRAS01866_0101 Ceratosolen vechti
S JRAS01866_0195 Ceratosolen vechti
I JRAS05491_0155 Ceratosolen nugatorius
S JRAS05491_0199 Ceratosolen nugatorius
I JRAS05491_0151 Ceratosolen nugatorius
I JRAS05491_0156 Ceratosolen nugatorius
I JRAS03020_0155 Ceratosolen nsp026
S JRAS03020_0195 Ceratosolen nsp026
I JRAS03020_0156 Ceratosolen nsp026
S JRAS03020_0102 Ceratosolen nsp026
S JRAS03020_0101 Ceratosolen nsp026
S JRAS01989_0195 Ceratosolen vetustus
I JRAS03016_0155 Ceratosolen vetustus
I JRAS03016_0156 Ceratosolen vetustus
S JRAS01989_0101 Ceratosolen vetustus
I JRAS03505_0151 Ceratosolen nsp035
S JRAS03507_0199 Ceratosolen nsp035
S JRAS03505_0101 Ceratosolen nsp035
I JRAS03505_0155 Ceratosolen nsp035
I JRAS03505_0156 Ceratosolen nsp035
S JRAS02043_0195 Ceratosolen nsp008
S JRAS02043_0101 Ceratosolen nsp008
I JRAS03564_0155 Ceratosolen nsp020
S JRAS03564_0101 Ceratosolen nsp020
I JRAS03564_0156 Ceratosolen nsp020
I JRAS03564_0151 Ceratosolen nsp020
S JRAS05478_0199 Ceratosolen nsp021
I JRAS05478_0155 Ceratosolen nsp021
S JRAS05478_0101 Ceratosolen nsp021
I JRAS05478_0151 Ceratosolen nsp021
I JRAS05478_0156 Ceratosolen nsp021
S JRAS01446_0101 Ceratosolen nsp018
S JRAS01446_0195 Ceratosolen nsp018
I JRAS01446_0155 Ceratosolen nsp018
I JRAS01446_0156 Ceratosolen nsp018
I JRAS01446_0151 Ceratosolen nsp018
I JRAS00213_0151 Ceratosolen nsp023

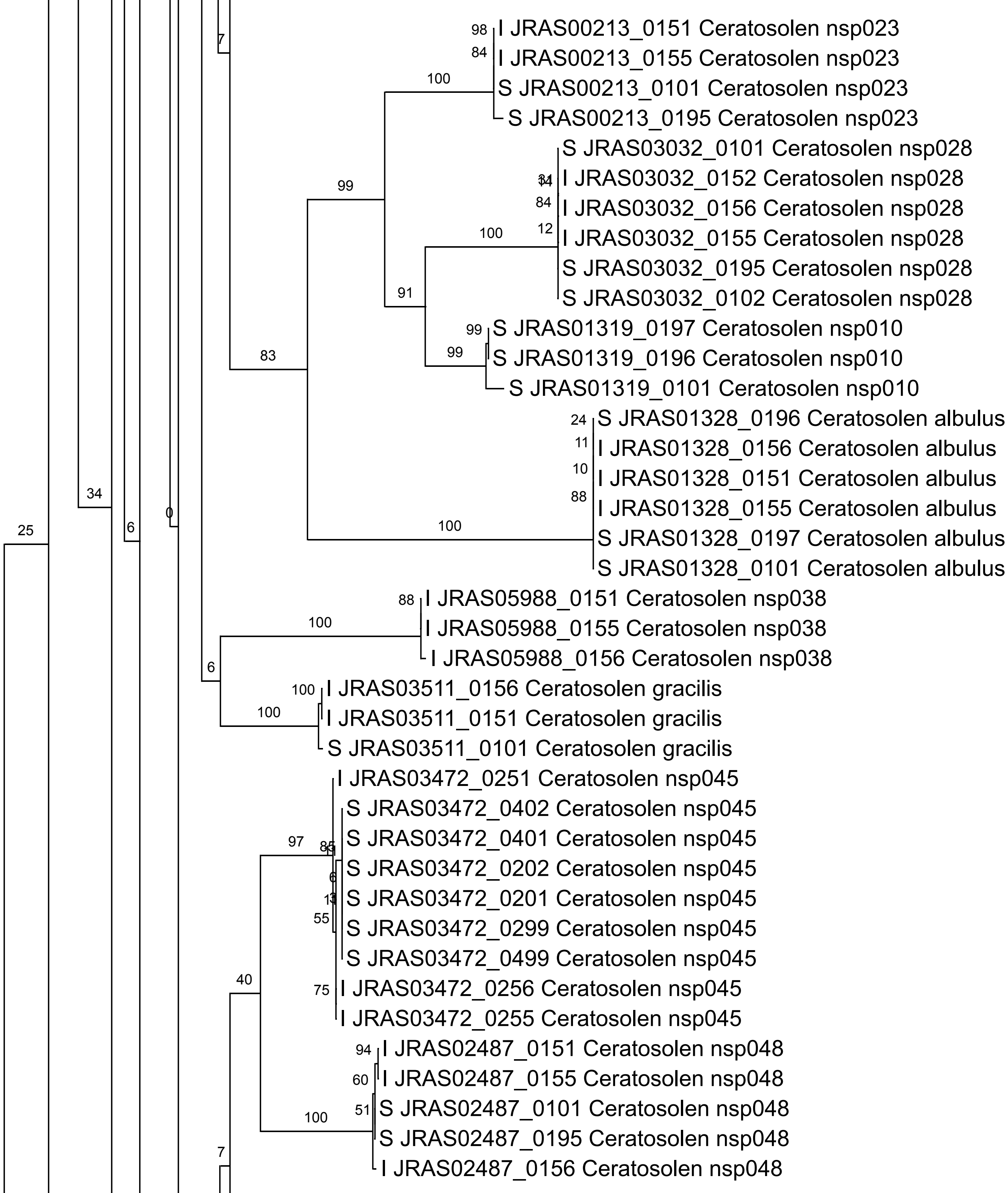
I JRAS00213_0151 Ceratosolen nsp023
I JRAS00213_0155 Ceratosolen nsp023
S JRAS00213_0101 Ceratosolen nsp023
S JRAS00213_0195 Ceratosolen nsp023
S JRAS03032_0101 Ceratosolen nsp028
I JRAS03032_0152 Ceratosolen nsp028
I JRAS03032_0156 Ceratosolen nsp028
I JRAS03032_0155 Ceratosolen nsp028
S JRAS03032_0195 Ceratosolen nsp028
S JRAS03032_0102 Ceratosolen nsp028
S JRAS01319_0197 Ceratosolen nsp010
S JRAS01319_0196 Ceratosolen nsp010
S JRAS01319_0101 Ceratosolen nsp010
S JRAS01328_0196 Ceratosolen albulus
I JRAS01328_0156 Ceratosolen albulus
I JRAS01328_0151 Ceratosolen albulus
I JRAS01328_0155 Ceratosolen albulus
S JRAS01328_0197 Ceratosolen albulus
S JRAS01328_0101 Ceratosolen albulus
I JRAS05988_0151 Ceratosolen nsp038
I JRAS05988_0155 Ceratosolen nsp038
I JRAS05988_0156 Ceratosolen nsp038
I JRAS03511_0156 Ceratosolen gracilis
I JRAS03511_0151 Ceratosolen gracilis
S JRAS03511_0101 Ceratosolen gracilis
I JRAS03472_0251 Ceratosolen nsp045
S JRAS03472_0402 Ceratosolen nsp045
S JRAS03472_0401 Ceratosolen nsp045
S JRAS03472_0202 Ceratosolen nsp045
S JRAS03472_0201 Ceratosolen nsp045
S JRAS03472_0299 Ceratosolen nsp045
S JRAS03472_0499 Ceratosolen nsp045
I JRAS03472_0256 Ceratosolen nsp045
I JRAS03472_0255 Ceratosolen nsp045
I JRAS02487_0151 Ceratosolen nsp048
I JRAS02487_0155 Ceratosolen nsp048
S JRAS02487_0101 Ceratosolen nsp048
S JRAS02487_0195 Ceratosolen nsp048
I JRAS02487_0156 Ceratosolen nsp048

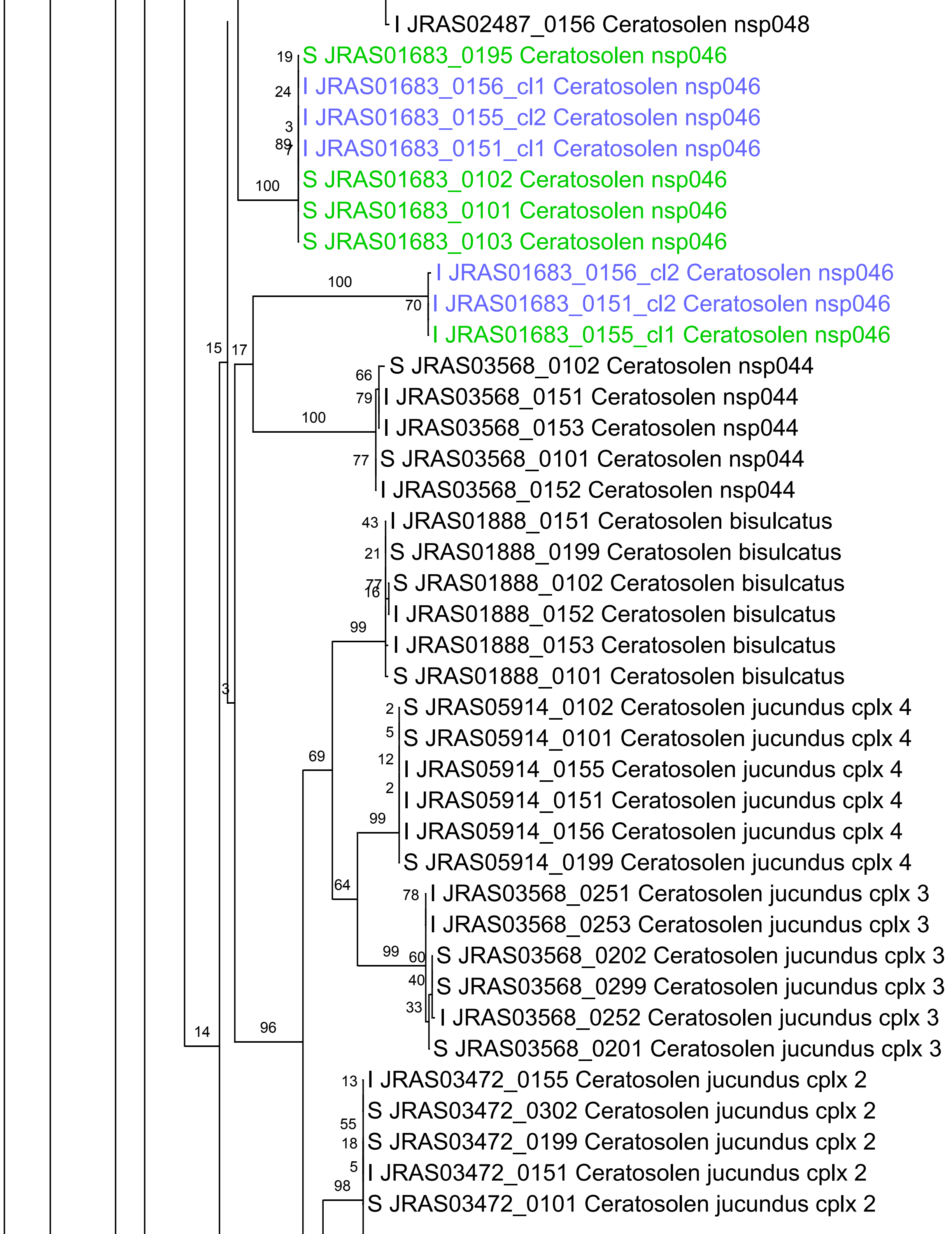
I JRAS02487_0156 Ceratosolen nsp048
S JRAS01683_0195 Ceratosolen nsp046
I JRAS01683_0156_cl1 Ceratosolen nsp046
I JRAS01683_0155_cl2 Ceratosolen nsp046
I JRAS01683_0151_cl1 Ceratosolen nsp046
S JRAS01683_0102 Ceratosolen nsp046
S JRAS01683_0101 Ceratosolen nsp046
S JRAS01683_0103 Ceratosolen nsp046
I JRAS01683_0156_cl2 Ceratosolen nsp046
I JRAS01683_0151_cl2 Ceratosolen nsp046
I JRAS01683_0155_cl1 Ceratosolen nsp046
S JRAS03568_0102 Ceratosolen nsp044
I JRAS03568_0151 Ceratosolen nsp044
I JRAS03568_0153 Ceratosolen nsp044
S JRAS03568_0101 Ceratosolen nsp044
I JRAS03568_0152 Ceratosolen nsp044
I JRAS01888_0151 Ceratosolen bisulcatus
S JRAS01888_0199 Ceratosolen bisulcatus
S JRAS01888_0102 Ceratosolen bisulcatus
I JRAS01888_0152 Ceratosolen bisulcatus
I JRAS01888_0153 Ceratosolen bisulcatus
S JRAS01888_0101 Ceratosolen bisulcatus
S JRAS05914_0102 Ceratosolen jucundus cplx 4
S JRAS05914_0101 Ceratosolen jucundus cplx 4
I JRAS05914_0155 Ceratosolen jucundus cplx 4
I JRAS05914_0151 Ceratosolen jucundus cplx 4
I JRAS05914_0156 Ceratosolen jucundus cplx 4
S JRAS05914_0199 Ceratosolen jucundus cplx 4
I JRAS03568_0251 Ceratosolen jucundus cplx 3
I JRAS03568_0253 Ceratosolen jucundus cplx 3
S JRAS03568_0202 Ceratosolen jucundus cplx 3
S JRAS03568_0299 Ceratosolen jucundus cplx 3
I JRAS03568_0252 Ceratosolen jucundus cplx 3
S JRAS03568_0201 Ceratosolen jucundus cplx 3
I JRAS03472_0155 Ceratosolen jucundus cplx 2
S JRAS03472_0302 Ceratosolen jucundus cplx 2
S JRAS03472_0199 Ceratosolen jucundus cplx 2
I JRAS03472_0151 Ceratosolen jucundus cplx 2
S JRAS03472_0101 Ceratosolen jucundus cplx 2

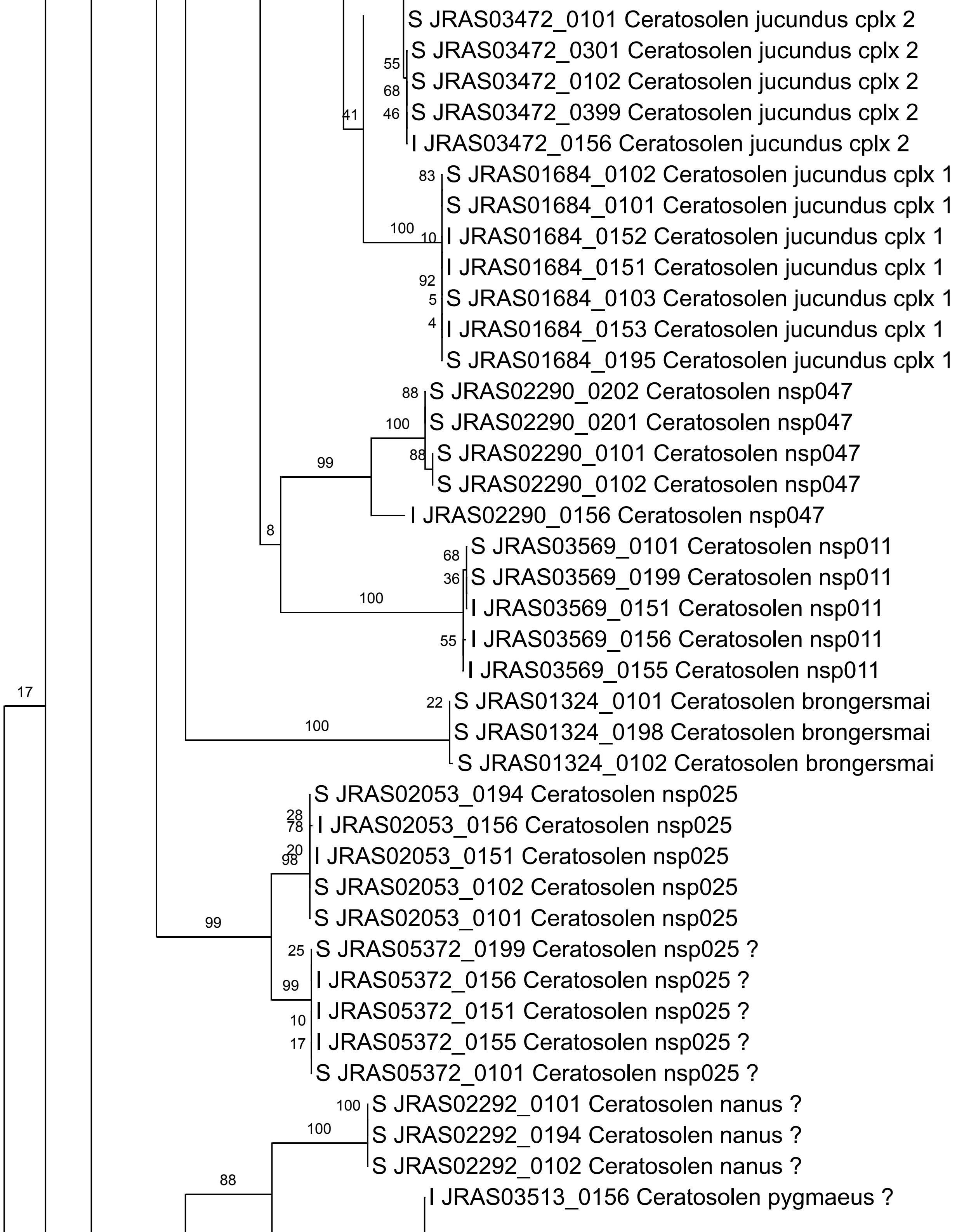
S JRAS03472_0101 Ceratosolen jucundus cplx 2
S JRAS03472_0301 Ceratosolen jucundus cplx 2
S JRAS03472_0102 Ceratosolen jucundus cplx 2
S JRAS03472_0399 Ceratosolen jucundus cplx 2
I JRAS03472_0156 Ceratosolen jucundus cplx 2
S JRAS01684_0102 Ceratosolen jucundus cplx 1
S JRAS01684_0101 Ceratosolen jucundus cplx 1
I JRAS01684_0152 Ceratosolen jucundus cplx 1
I JRAS01684_0151 Ceratosolen jucundus cplx 1
S JRAS01684_0103 Ceratosolen jucundus cplx 1
I JRAS01684_0153 Ceratosolen jucundus cplx 1
S JRAS01684_0195 Ceratosolen jucundus cplx 1
S JRAS02290_0202 Ceratosolen nsp047
S JRAS02290_0201 Ceratosolen nsp047
S JRAS02290_0101 Ceratosolen nsp047
S JRAS02290_0102 Ceratosolen nsp047
I JRAS02290_0156 Ceratosolen nsp047
S JRAS03569_0101 Ceratosolen nsp011
S JRAS03569_0199 Ceratosolen nsp011
I JRAS03569_0151 Ceratosolen nsp011
I JRAS03569_0156 Ceratosolen nsp011
I JRAS03569_0155 Ceratosolen nsp011
S JRAS01324_0101 Ceratosolen brongersmai
S JRAS01324_0198 Ceratosolen brongersmai
S JRAS01324_0102 Ceratosolen brongersmai
S JRAS02053_0194 Ceratosolen nsp025
I JRAS02053_0156 Ceratosolen nsp025
I JRAS02053_0151 Ceratosolen nsp025
S JRAS02053_0102 Ceratosolen nsp025
S JRAS02053_0101 Ceratosolen nsp025
S JRAS05372_0199 Ceratosolen nsp025 ?
I JRAS05372_0156 Ceratosolen nsp025 ?
I JRAS05372_0151 Ceratosolen nsp025 ?
I JRAS05372_0155 Ceratosolen nsp025 ?
S JRAS05372_0101 Ceratosolen nsp025 ?
S JRAS02292_0101 Ceratosolen nanus ?
S JRAS02292_0194 Ceratosolen nanus ?
S JRAS02292_0102 Ceratosolen nanus ?
I JRAS03513_0156 Ceratosolen pygmaeus ?

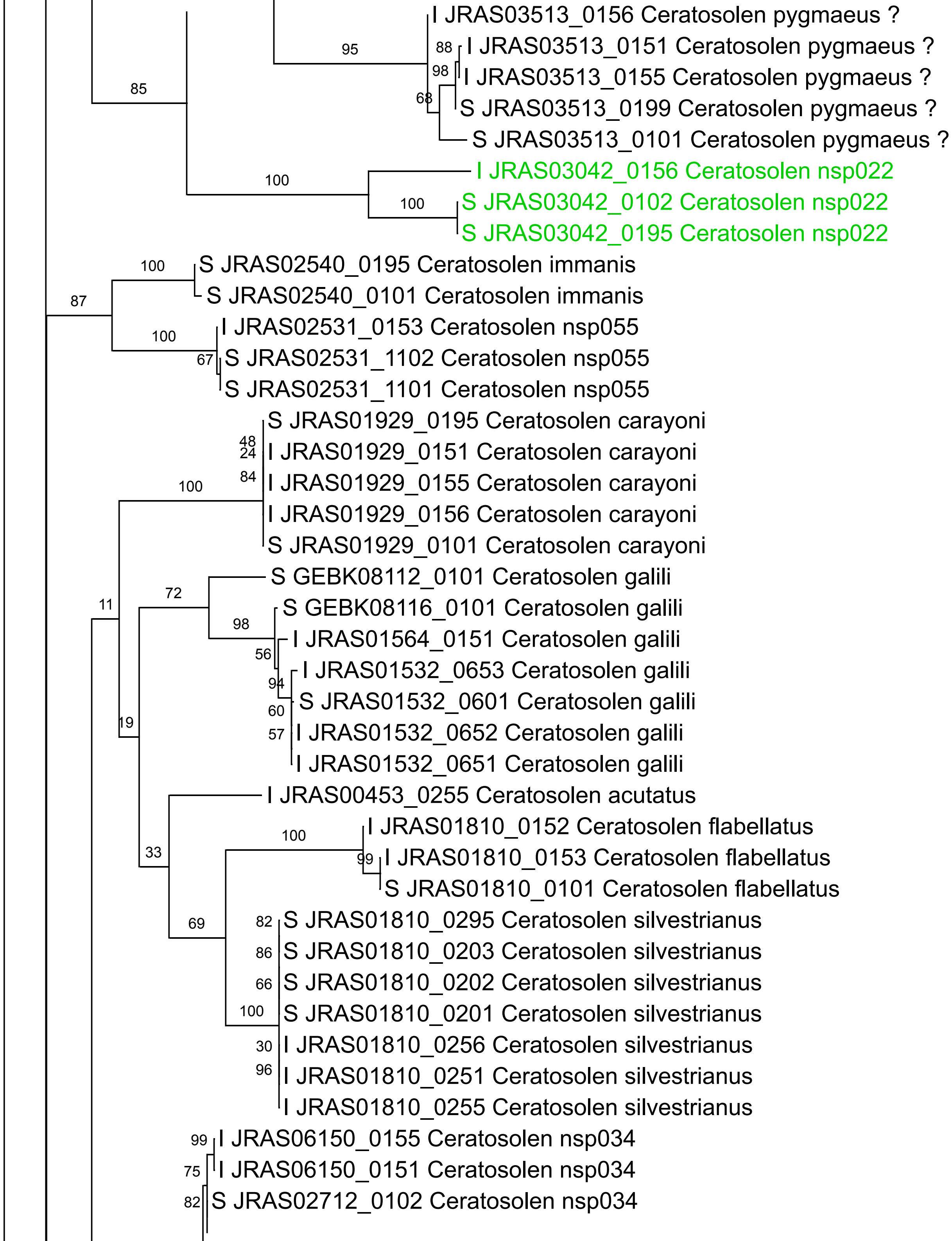
I JRAS03513_0156 Ceratosolen pygmaeus ?
I JRAS03513_0151 Ceratosolen pygmaeus ?
I JRAS03513_0155 Ceratosolen pygmaeus ?
S JRAS03513_0199 Ceratosolen pygmaeus ?
S JRAS03513_0101 Ceratosolen pygmaeus ?
I JRAS03042_0156 Ceratosolen nsp022
S JRAS03042_0102 Ceratosolen nsp022
S JRAS03042_0195 Ceratosolen nsp022
S JRAS02540_0195 Ceratosolen immanis
S JRAS02540_0101 Ceratosolen immanis
I JRAS02531_0153 Ceratosolen nsp055
S JRAS02531_1102 Ceratosolen nsp055
S JRAS02531_1101 Ceratosolen nsp055
S JRAS01929_0195 Ceratosolen carayoni
I JRAS01929_0151 Ceratosolen carayoni
I JRAS01929_0155 Ceratosolen carayoni
I JRAS01929_0156 Ceratosolen carayoni
S JRAS01929_0101 Ceratosolen carayoni
S GEBK08112_0101 Ceratosolen galili
S GEBK08116_0101 Ceratosolen galili
I JRAS01564_0151 Ceratosolen galili
I JRAS01532_0653 Ceratosolen galili
S JRAS01532_0601 Ceratosolen galili
I JRAS01532_0652 Ceratosolen galili
I JRAS01532_0651 Ceratosolen galili
I JRAS00453_0255 Ceratosolen acutatus
I JRAS01810_0152 Ceratosolen flabellatus
I JRAS01810_0153 Ceratosolen flabellatus
S JRAS01810_0101 Ceratosolen flabellatus
S JRAS01810_0295 Ceratosolen silvestrianus
S JRAS01810_0203 Ceratosolen silvestrianus
S JRAS01810_0202 Ceratosolen silvestrianus
S JRAS01810_0201 Ceratosolen silvestrianus
I JRAS01810_0256 Ceratosolen silvestrianus
I JRAS01810_0251 Ceratosolen silvestrianus
I JRAS01810_0255 Ceratosolen silvestrianus
I JRAS06150_0155 Ceratosolen nsp034
I JRAS06150_0151 Ceratosolen nsp034
S JRAS02712_0102 Ceratosolen nsp034

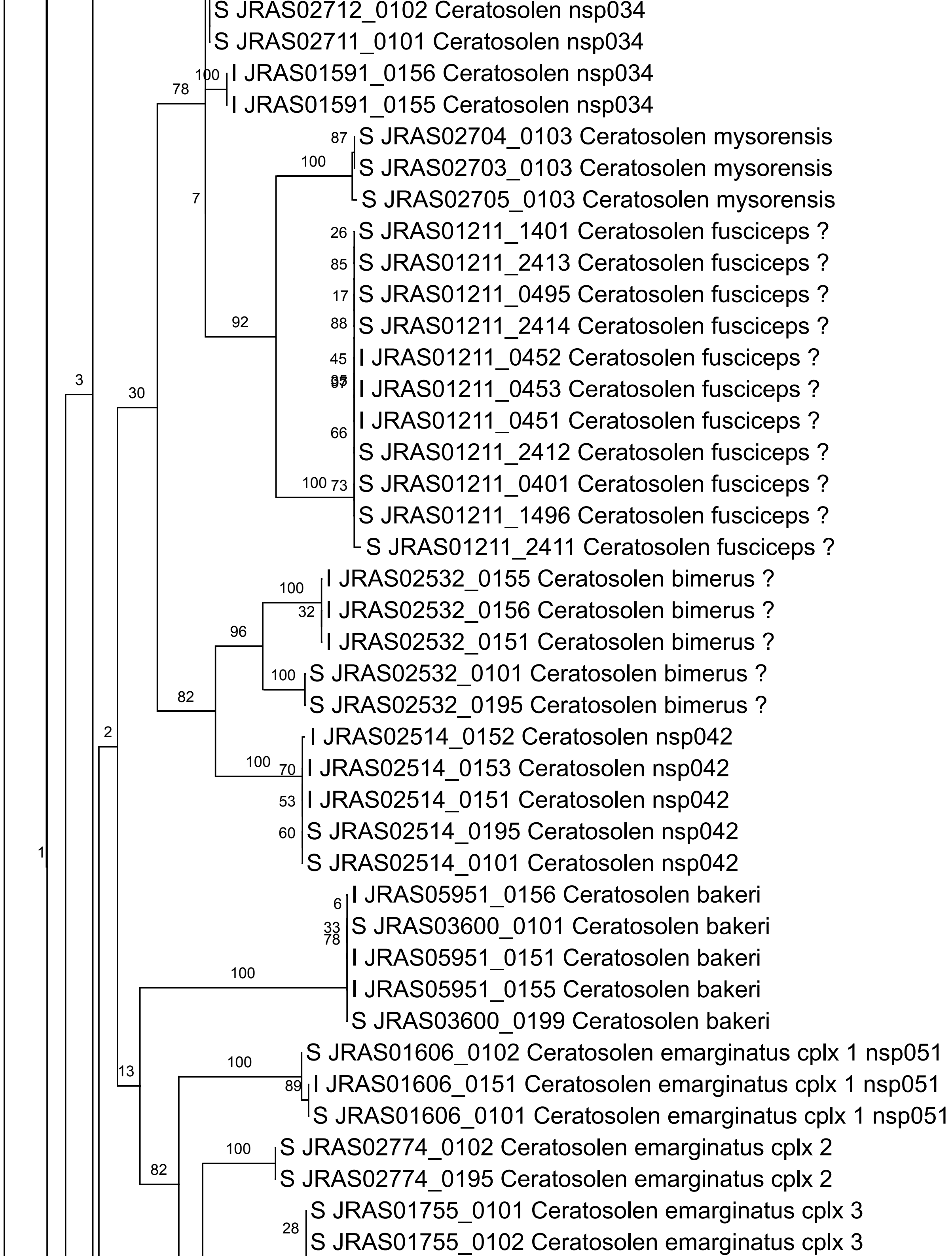
S JRAS02712_0102 Ceratosolen nsp034
S JRAS02711_0101 Ceratosolen nsp034
I JRAS01591_0156 Ceratosolen nsp034
I JRAS01591_0155 Ceratosolen nsp034
S JRAS02704_0103 Ceratosolen mysorensis
S JRAS02703_0103 Ceratosolen mysorensis
S JRAS02705_0103 Ceratosolen mysorensis
S JRAS01211_1401 Ceratosolen fusciceps ?
S JRAS01211_2413 Ceratosolen fusciceps ?
S JRAS01211_0495 Ceratosolen fusciceps ?
S JRAS01211_2414 Ceratosolen fusciceps ?
I JRAS01211_0452 Ceratosolen fusciceps ?
I JRAS01211_0453 Ceratosolen fusciceps ?
I JRAS01211_0451 Ceratosolen fusciceps ?
S JRAS01211_2412 Ceratosolen fusciceps ?
S JRAS01211_0401 Ceratosolen fusciceps ?
S JRAS01211_1496 Ceratosolen fusciceps ?
S JRAS01211_2411 Ceratosolen fusciceps ?
I JRAS02532_0155 Ceratosolen bimerus ?
I JRAS02532_0156 Ceratosolen bimerus ?
I JRAS02532_0151 Ceratosolen bimerus ?
S JRAS02532_0101 Ceratosolen bimerus ?
S JRAS02532_0195 Ceratosolen bimerus ?
I JRAS02514_0152 Ceratosolen nsp042
I JRAS02514_0153 Ceratosolen nsp042
I JRAS02514_0151 Ceratosolen nsp042
S JRAS02514_0195 Ceratosolen nsp042
S JRAS02514_0101 Ceratosolen nsp042
I JRAS05951_0156 Ceratosolen bakeri
S JRAS03600_0101 Ceratosolen bakeri
I JRAS05951_0151 Ceratosolen bakeri
I JRAS05951_0155 Ceratosolen bakeri
S JRAS03600_0199 Ceratosolen bakeri
S JRAS01606_0102 Ceratosolen emarginatus cplx 1 nsp051
I JRAS01606_0151 Ceratosolen emarginatus cplx 1 nsp051
S JRAS01606_0101 Ceratosolen emarginatus cplx 1 nsp051
S JRAS02774_0102 Ceratosolen emarginatus cplx 2
S JRAS02774_0195 Ceratosolen emarginatus cplx 2
S JRAS01755_0101 Ceratosolen emarginatus cplx 3
S JRAS01755_0102 Ceratosolen emarginatus cplx 3

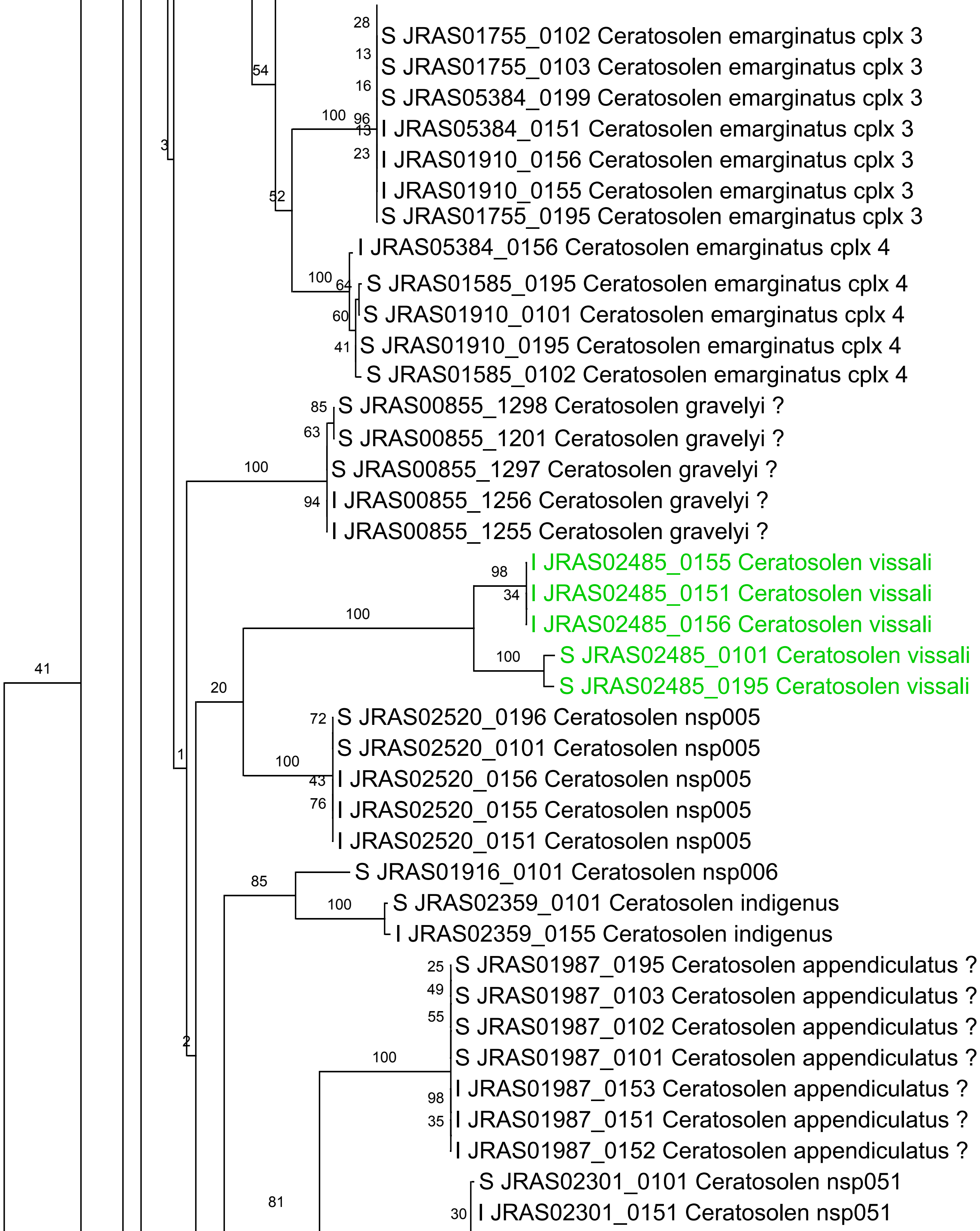
S JRAS01755_0102 Ceratosolen emarginatus cplx 3
S JRAS01755_0103 Ceratosolen emarginatus cplx 3
S JRAS05384_0199 Ceratosolen emarginatus cplx 3
I JRAS05384_0151 Ceratosolen emarginatus cplx 3
I JRAS01910_0156 Ceratosolen emarginatus cplx 3
I JRAS01910_0155 Ceratosolen emarginatus cplx 3
S JRAS01755_0195 Ceratosolen emarginatus cplx 3
I JRAS05384_0156 Ceratosolen emarginatus cplx 4
S JRAS01585_0195 Ceratosolen emarginatus cplx 4
S JRAS01910_0101 Ceratosolen emarginatus cplx 4
S JRAS01910_0195 Ceratosolen emarginatus cplx 4
S JRAS01585_0102 Ceratosolen emarginatus cplx 4
S JRAS00855_1298 Ceratosolen gravelyi ?
S JRAS00855_1201 Ceratosolen gravelyi ?
S JRAS00855_1297 Ceratosolen gravelyi ?
I JRAS00855_1256 Ceratosolen gravelyi ?
I JRAS00855_1255 Ceratosolen gravelyi ?
I JRAS02485_0155 Ceratosolen vissali
I JRAS02485_0151 Ceratosolen vissali
I JRAS02485_0156 Ceratosolen vissali
S JRAS02485_0101 Ceratosolen vissali
S JRAS02485_0195 Ceratosolen vissali
S JRAS02520_0196 Ceratosolen nsp005
S JRAS02520_0101 Ceratosolen nsp005
I JRAS02520_0156 Ceratosolen nsp005
I JRAS02520_0155 Ceratosolen nsp005
I JRAS02520_0151 Ceratosolen nsp005
S JRAS01916_0101 Ceratosolen nsp006
S JRAS02359_0101 Ceratosolen indigenus
I JRAS02359_0155 Ceratosolen indigenus
S JRAS01987_0195 Ceratosolen appendiculatus ?
S JRAS01987_0103 Ceratosolen appendiculatus ?
S JRAS01987_0102 Ceratosolen appendiculatus ?
S JRAS01987_0101 Ceratosolen appendiculatus ?
I JRAS01987_0153 Ceratosolen appendiculatus ?
I JRAS01987_0151 Ceratosolen appendiculatus ?
I JRAS01987_0152 Ceratosolen appendiculatus ?
S JRAS02301_0101 Ceratosolen nsp051
I JRAS02301_0151 Ceratosolen nsp051

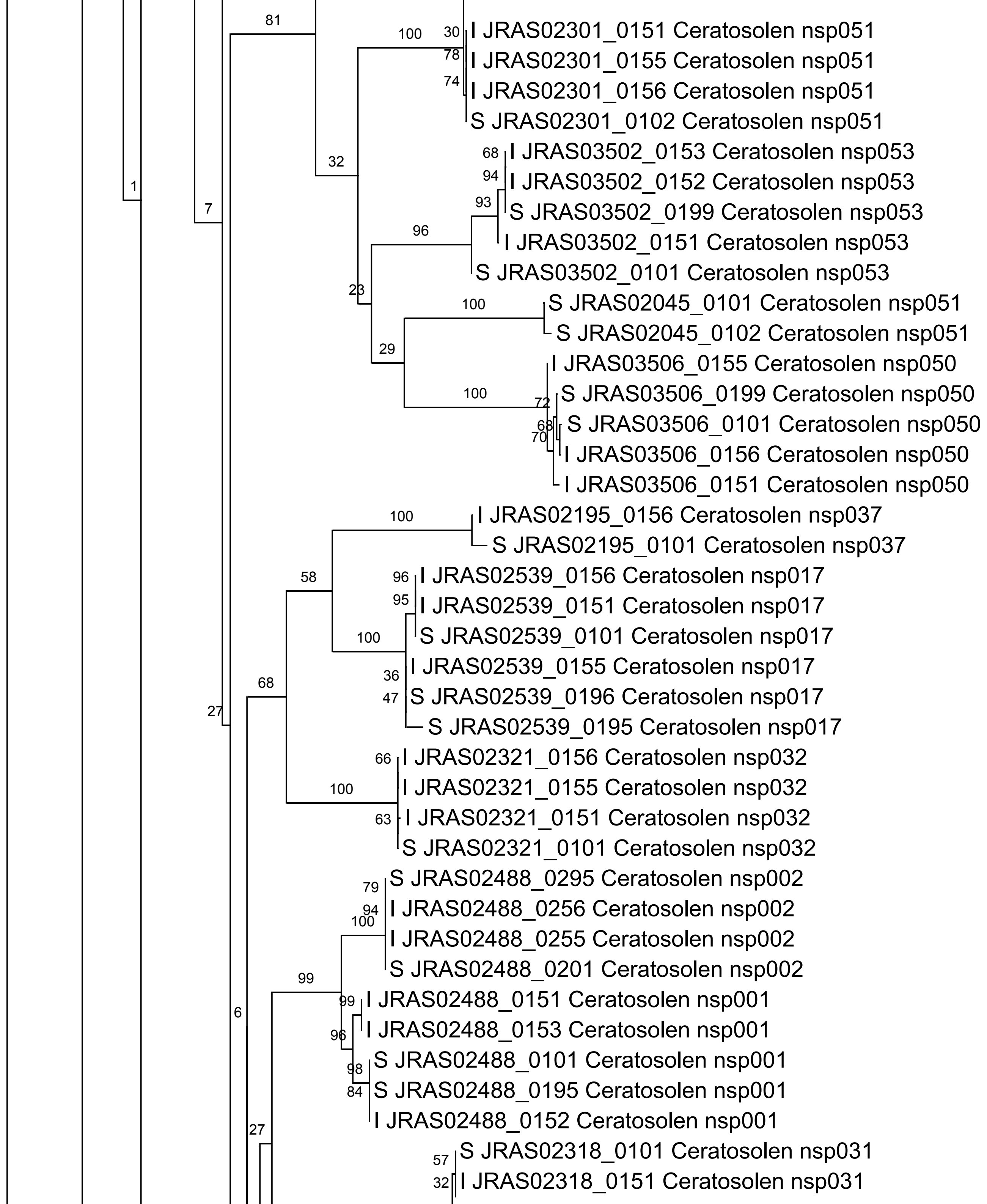
I JRAS02301_0151 Ceratosolen nsp051
I JRAS02301_0155 Ceratosolen nsp051
I JRAS02301_0156 Ceratosolen nsp051
S JRAS02301_0102 Ceratosolen nsp051
I JRAS03502_0153 Ceratosolen nsp053
I JRAS03502_0152 Ceratosolen nsp053
S JRAS03502_0199 Ceratosolen nsp053
I JRAS03502_0151 Ceratosolen nsp053
S JRAS03502_0101 Ceratosolen nsp053
S JRAS02045_0101 Ceratosolen nsp051
S JRAS02045_0102 Ceratosolen nsp051
I JRAS03506_0155 Ceratosolen nsp050
S JRAS03506_0199 Ceratosolen nsp050
S JRAS03506_0101 Ceratosolen nsp050
I JRAS03506_0156 Ceratosolen nsp050
I JRAS03506_0151 Ceratosolen nsp050
I JRAS02195_0156 Ceratosolen nsp037
S JRAS02195_0101 Ceratosolen nsp037
I JRAS02539_0156 Ceratosolen nsp017
I JRAS02539_0151 Ceratosolen nsp017
S JRAS02539_0101 Ceratosolen nsp017
I JRAS02539_0155 Ceratosolen nsp017
S JRAS02539_0196 Ceratosolen nsp017
S JRAS02539_0195 Ceratosolen nsp017
I JRAS02321_0156 Ceratosolen nsp032
I JRAS02321_0155 Ceratosolen nsp032
I JRAS02321_0151 Ceratosolen nsp032
S JRAS02321_0101 Ceratosolen nsp032
S JRAS02488_0295 Ceratosolen nsp002
I JRAS02488_0256 Ceratosolen nsp002
I JRAS02488_0255 Ceratosolen nsp002
S JRAS02488_0201 Ceratosolen nsp002
I JRAS02488_0151 Ceratosolen nsp001
I JRAS02488_0153 Ceratosolen nsp001
S JRAS02488_0101 Ceratosolen nsp001
S JRAS02488_0195 Ceratosolen nsp001
I JRAS02488_0152 Ceratosolen nsp001
S JRAS02318_0101 Ceratosolen nsp031
I JRAS02318_0151 Ceratosolen nsp031

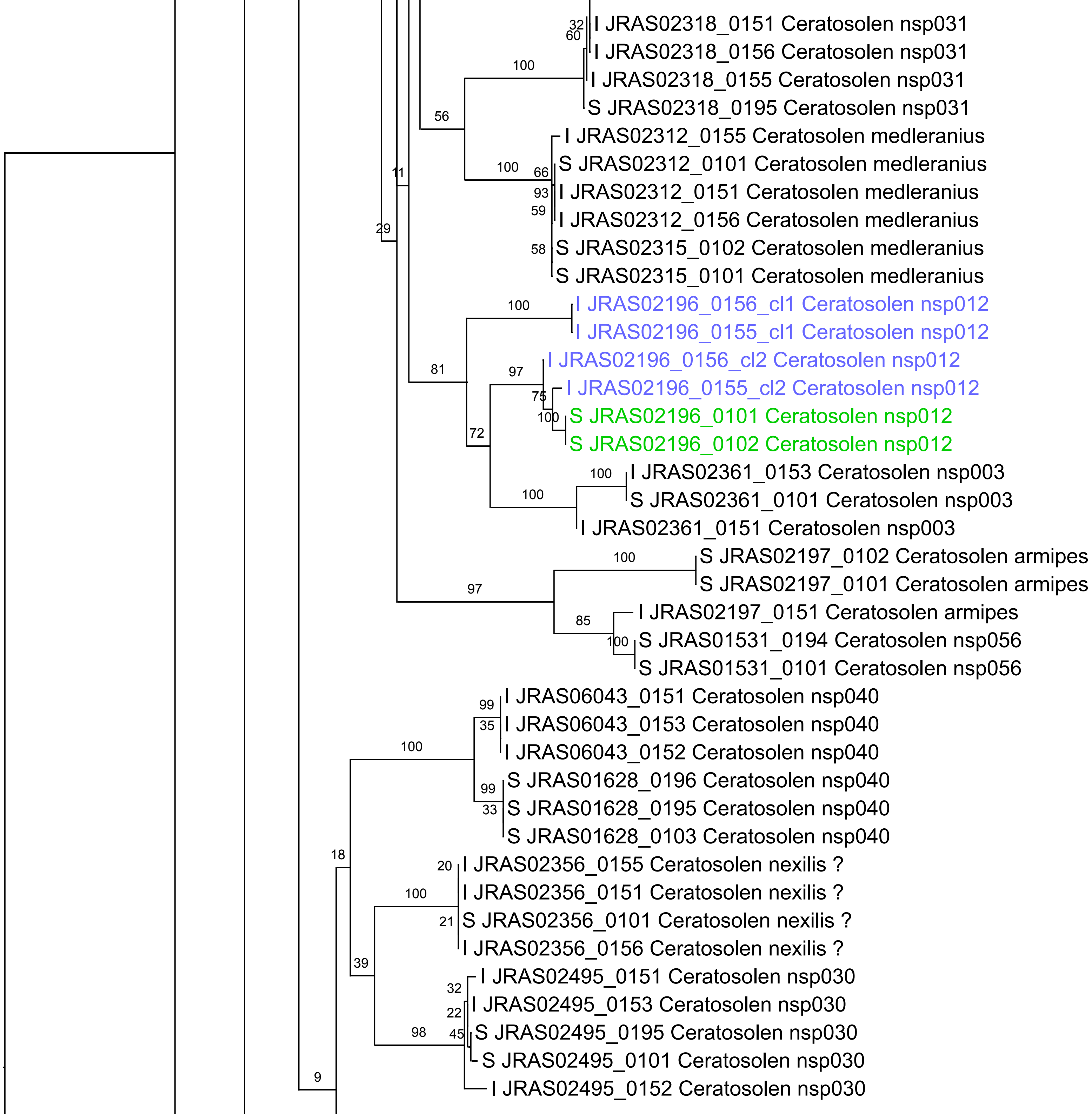

I JRAS02318_0151 Ceratosolen nsp031
I JRAS02318_0156 Ceratosolen nsp031
I JRAS02318_0155 Ceratosolen nsp031
S JRAS02318_0195 Ceratosolen nsp031
I JRAS02312_0155 Ceratosolen medleranius
S JRAS02312_0101 Ceratosolen medleranius
I JRAS02312_0151 Ceratosolen medleranius
I JRAS02312_0156 Ceratosolen medleranius
S JRAS02315_0102 Ceratosolen medleranius
S JRAS02315_0101 Ceratosolen medleranius
I JRAS02196_0156_cl1 Ceratosolen nsp012
I JRAS02196_0155_cl1 Ceratosolen nsp012
I JRAS02196_0156_cl2 Ceratosolen nsp012
I JRAS02196_0155_cl2 Ceratosolen nsp012
S JRAS02196_0101 Ceratosolen nsp012
S JRAS02196_0102 Ceratosolen nsp012
I JRAS02361_0153 Ceratosolen nsp003
S JRAS02361_0101 Ceratosolen nsp003
I JRAS02361_0151 Ceratosolen nsp003
S JRAS02197_0102 Ceratosolen armipes
S JRAS02197_0101 Ceratosolen armipes
I JRAS02197_0151 Ceratosolen armipes
S JRAS01531_0194 Ceratosolen nsp056
S JRAS01531_0101 Ceratosolen nsp056
I JRAS06043_0151 Ceratosolen nsp040
I JRAS06043_0153 Ceratosolen nsp040
I JRAS06043_0152 Ceratosolen nsp040
S JRAS01628_0196 Ceratosolen nsp040
S JRAS01628_0195 Ceratosolen nsp040
S JRAS01628_0103 Ceratosolen nsp040
I JRAS02356_0155 Ceratosolen nexilis ?
I JRAS02356_0151 Ceratosolen nexilis ?
S JRAS02356_0101 Ceratosolen nexilis ?
I JRAS02356_0156 Ceratosolen nexilis ?
I JRAS02495_0151 Ceratosolen nsp030
I JRAS02495_0153 Ceratosolen nsp030
S JRAS02495_0195 Ceratosolen nsp030
S JRAS02495_0101 Ceratosolen nsp030
I JRAS02495_0152 Ceratosolen nsp030

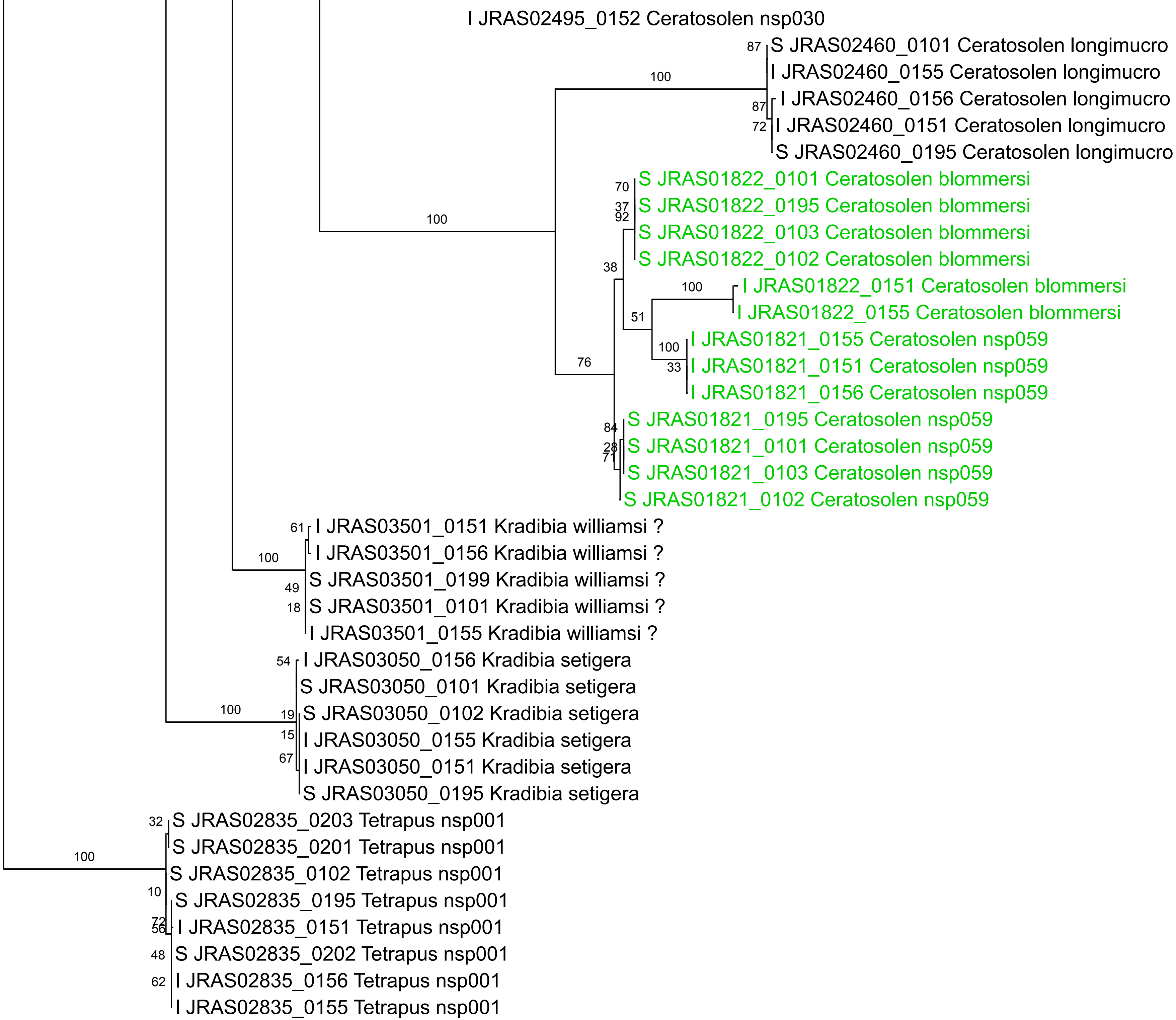
I JRAS02495_0152 Ceratosolen nsp030
87
S JRAS02460_0101 Ceratosolen longimucro
100
I JRAS02460_0155 Ceratosolen longimucro
87
I JRAS02460_0156 Ceratosolen longimucro
72
I JRAS02460_0151 Ceratosolen longimucro
S JRAS02460_0195 Ceratosolen longimucro
70
S JRAS01822_0101 Ceratosolen blommersi
37
92
S JRAS01822_0195 Ceratosolen blommersi
100
S JRAS01822_0103 Ceratosolen blommersi
S JRAS01822_0102 Ceratosolen blommersi
38
100
I JRAS01822_0151 Ceratosolen blommersi
51
I JRAS01822_0155 Ceratosolen blommersi
100
I JRAS01821_0155 Ceratosolen nsp059
76
33
I JRAS01821_0151 Ceratosolen nsp059
I JRAS01821_0156 Ceratosolen nsp059
84
S JRAS01821_0195 Ceratosolen nsp059
28
71
S JRAS01821_0101 Ceratosolen nsp059
S JRAS01821_0103 Ceratosolen nsp059
S JRAS01821_0102 Ceratosolen nsp059
61
I JRAS03501_0151 Kradibia williamsi ?
100
I JRAS03501_0156 Kradibia williamsi ?
49
S JRAS03501_0199 Kradibia williamsi ?
18
S JRAS03501_0101 Kradibia williamsi ?
I JRAS03501_0155 Kradibia williamsi ?
54
I JRAS03050_0156 Kradibia setigera
S JRAS03050_0101 Kradibia setigera
100
19
S JRAS03050_0102 Kradibia setigera
15
I JRAS03050_0155 Kradibia setigera
67
I JRAS03050_0151 Kradibia setigera
S JRAS03050_0195 Kradibia setigera
32
S JRAS02835_0203 Tetrapus nsp001
100
S JRAS02835_0201 Tetrapus nsp001
S JRAS02835_0102 Tetrapus nsp001
10
S JRAS02835_0195 Tetrapus nsp001
72
56
I JRAS02835_0151 Tetrapus nsp001
48
S JRAS02835_0202 Tetrapus nsp001
62
I JRAS02835_0156 Tetrapus nsp001
I JRAS02835_0155 Tetrapus nsp001

Fig S3. RAxML tree for the EF data set (Miseq+Sanger) (BP : 1000 replicates).

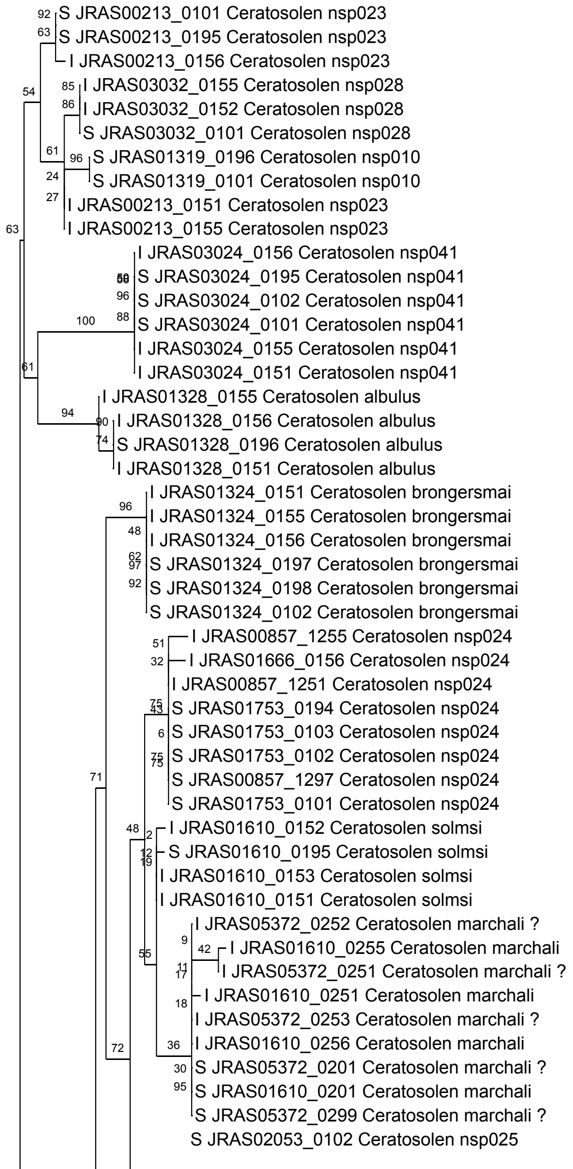

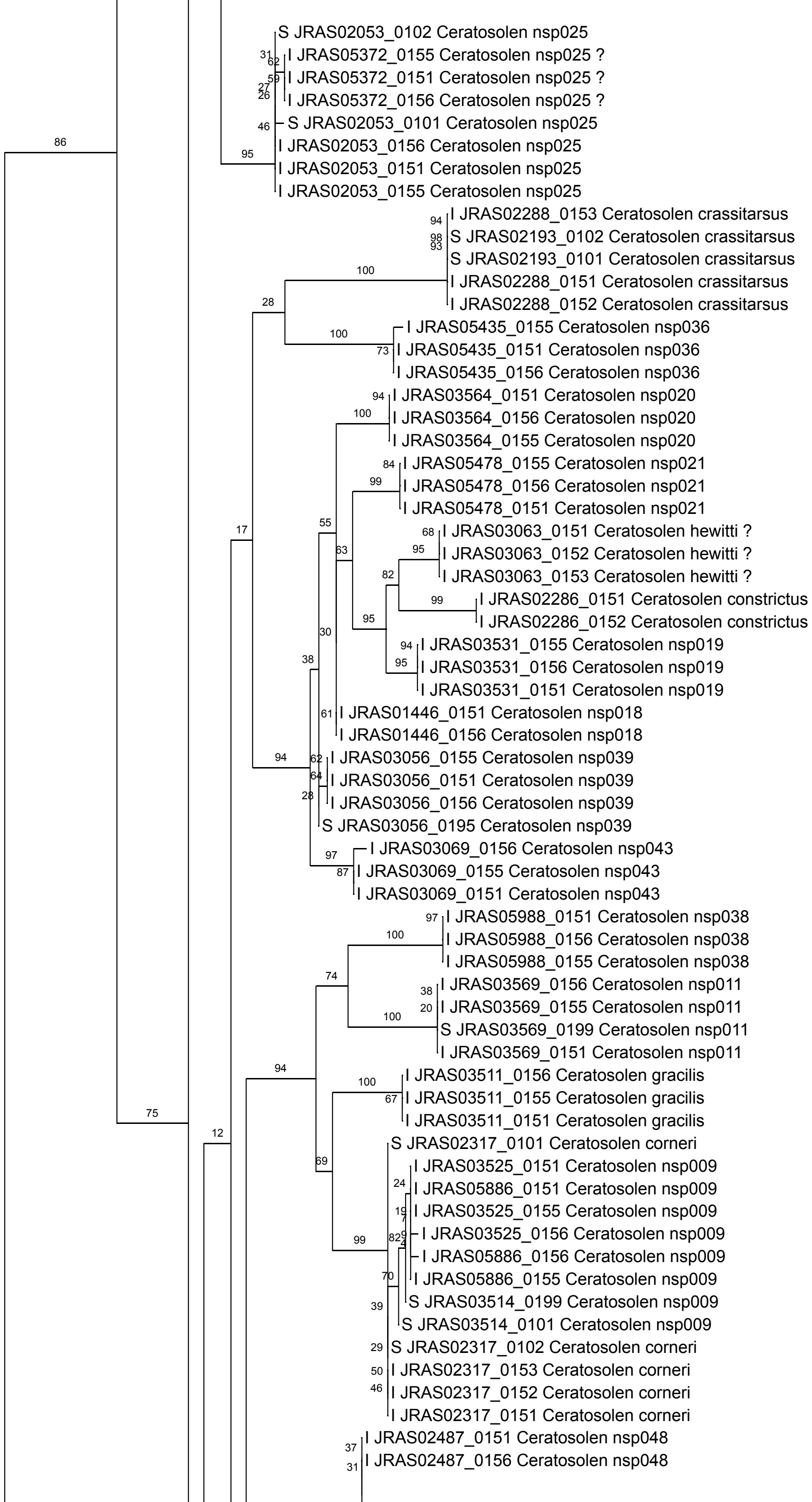

S JRAS02053_0102 Ceratosolen nsp025
I JRAS05372_0155 Ceratosolen nsp025 ?
I JRAS05372_0151 Ceratosolen nsp025 ?
I JRAS05372_0156 Ceratosolen nsp025 ?
S JRAS02053_0101 Ceratosolen nsp025
I JRAS02053_0156 Ceratosolen nsp025
I JRAS02053_0151 Ceratosolen nsp025
I JRAS02053_0155 Ceratosolen nsp025
I JRAS02288_0153 Ceratosolen crassitarsus
S JRAS02193_0102 Ceratosolen crassitarsus
S JRAS02193_0101 Ceratosolen crassitarsus
I JRAS02288_0151 Ceratosolen crassitarsus
I JRAS02288_0152 Ceratosolen crassitarsus
I JRAS05435_0155 Ceratosolen nsp036
I JRAS05435_0151 Ceratosolen nsp036
I JRAS05435_0156 Ceratosolen nsp036
I JRAS03564_0151 Ceratosolen nsp020
I JRAS03564_0156 Ceratosolen nsp020
I JRAS03564_0155 Ceratosolen nsp020
I JRAS05478_0155 Ceratosolen nsp021
I JRAS05478_0156 Ceratosolen nsp021
I JRAS05478_0151 Ceratosolen nsp021
I JRAS03063_0151 Ceratosolen hewitti ?
I JRAS03063_0152 Ceratosolen hewitti ?
I JRAS03063_0153 Ceratosolen hewitti ?
I JRAS02286_0151 Ceratosolen constrictus
I JRAS02286_0152 Ceratosolen constrictus
I JRAS03531_0155 Ceratosolen nsp019
I JRAS03531_0156 Ceratosolen nsp019
I JRAS03531_0151 Ceratosolen nsp019
I JRAS01446_0151 Ceratosolen nsp018
I JRAS01446_0156 Ceratosolen nsp018
I JRAS03056_0155 Ceratosolen nsp039
I JRAS03056_0151 Ceratosolen nsp039
I JRAS03056_0156 Ceratosolen nsp039
S JRAS03056_0195 Ceratosolen nsp039
I JRAS03069_0156 Ceratosolen nsp043
I JRAS03069_0155 Ceratosolen nsp043
I JRAS03069_0151 Ceratosolen nsp043
I JRAS05988_0151 Ceratosolen nsp038
I JRAS05988_0156 Ceratosolen nsp038
I JRAS05988_0155 Ceratosolen nsp038
I JRAS03569_0156 Ceratosolen nsp011
I JRAS03569_0155 Ceratosolen nsp011
S JRAS03569_0199 Ceratosolen nsp011
I JRAS03569_0151 Ceratosolen nsp011
I JRAS03511_0156 Ceratosolen gracilis
I JRAS03511_0155 Ceratosolen gracilis
I JRAS03511_0151 Ceratosolen gracilis
S JRAS02317_0101 Ceratosolen corneri
I JRAS03525_0151 Ceratosolen nsp009
I JRAS05886_0151 Ceratosolen nsp009
I JRAS03525_0155 Ceratosolen nsp009
I JRAS03525_0156 Ceratosolen nsp009
I JRAS05886_0156 Ceratosolen nsp009
I JRAS05886_0155 Ceratosolen nsp009
S JRAS03514_0199 Ceratosolen nsp009
S JRAS03514_0101 Ceratosolen nsp009
S JRAS02317_0102 Ceratosolen corneri
I JRAS02317_0153 Ceratosolen corneri
I JRAS02317_0152 Ceratosolen corneri
I JRAS02317_0151 Ceratosolen corneri
I JRAS02487_0151 Ceratosolen nsp048
I JRAS02487_0156 Ceratosolen nsp048

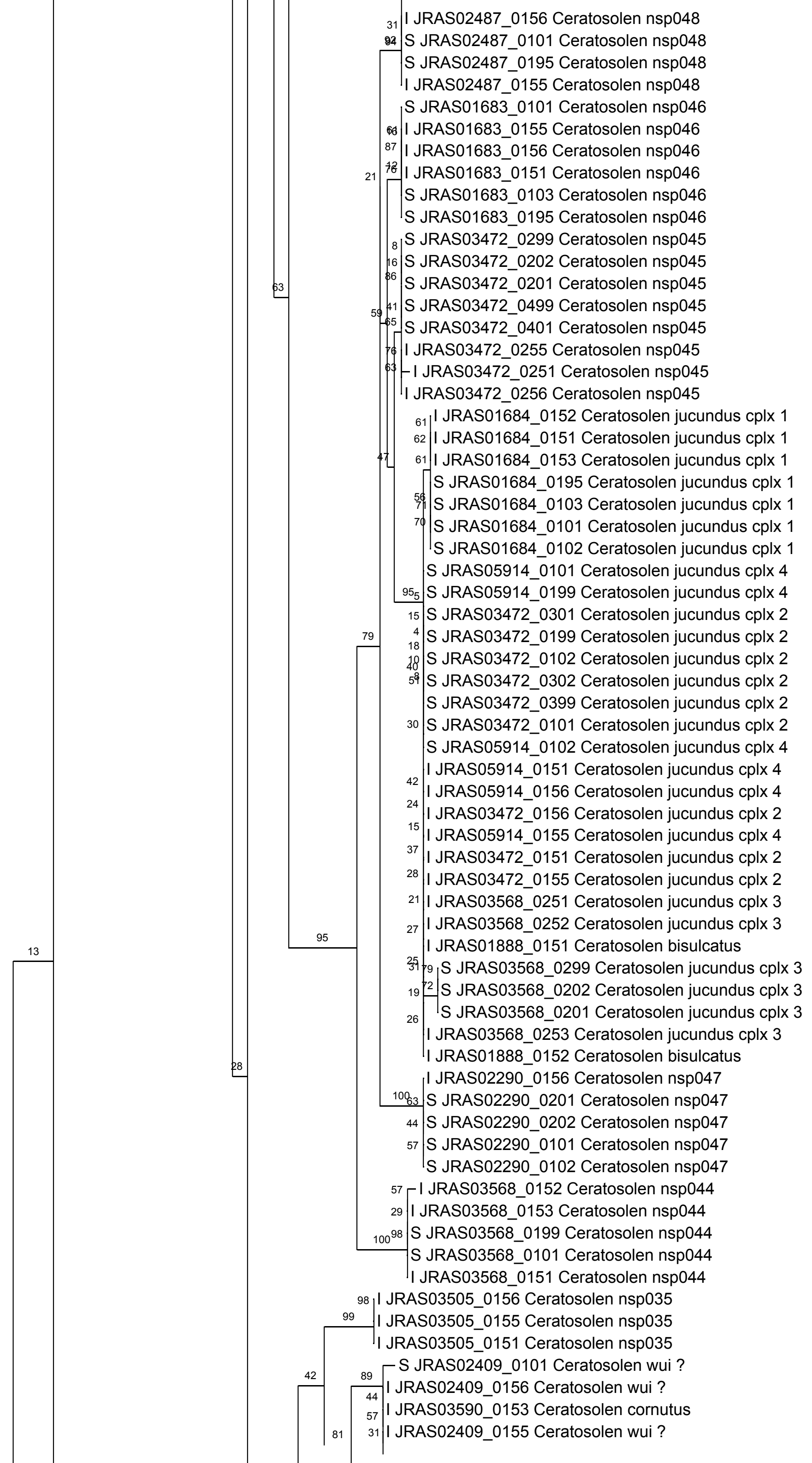

I JRAS02487_0156 Ceratosolen nsp048
S JRAS02487_0101 Ceratosolen nsp048
S JRAS02487_0195 Ceratosolen nsp048
I JRAS02487_0155 Ceratosolen nsp048
S JRAS01683_0101 Ceratosolen nsp046
I JRAS01683_0155 Ceratosolen nsp046
I JRAS01683_0156 Ceratosolen nsp046
I JRAS01683_0151 Ceratosolen nsp046
S JRAS01683_0103 Ceratosolen nsp046
S JRAS01683_0195 Ceratosolen nsp046
S JRAS03472_0299 Ceratosolen nsp045
S JRAS03472_0202 Ceratosolen nsp045
S JRAS03472_0201 Ceratosolen nsp045
S JRAS03472_0499 Ceratosolen nsp045
S JRAS03472_0401 Ceratosolen nsp045
I JRAS03472_0255 Ceratosolen nsp045
I JRAS03472_0251 Ceratosolen nsp045
I JRAS03472_0256 Ceratosolen nsp045
I JRAS01684_0152 Ceratosolen jucundus cplx 1
I JRAS01684_0151 Ceratosolen jucundus cplx 1
I JRAS01684_0153 Ceratosolen jucundus cplx 1
S JRAS01684_0195 Ceratosolen jucundus cplx 1
S JRAS01684_0103 Ceratosolen jucundus cplx 1
S JRAS01684_0101 Ceratosolen jucundus cplx 1
S JRAS01684_0102 Ceratosolen jucundus cplx 1
S JRAS05914_0101 Ceratosolen jucundus cplx 4
S JRAS05914_0199 Ceratosolen jucundus cplx 4
S JRAS03472_0301 Ceratosolen jucundus cplx 2
S JRAS03472_0199 Ceratosolen jucundus cplx 2
S JRAS03472_0102 Ceratosolen jucundus cplx 2
S JRAS03472_0302 Ceratosolen jucundus cplx 2
S JRAS03472_0399 Ceratosolen jucundus cplx 2
S JRAS03472_0101 Ceratosolen jucundus cplx 2
S JRAS05914_0102 Ceratosolen jucundus cplx 4
I JRAS05914_0151 Ceratosolen jucundus cplx 4
I JRAS05914_0156 Ceratosolen jucundus cplx 4
I JRAS03472_0156 Ceratosolen jucundus cplx 2
I JRAS05914_0155 Ceratosolen jucundus cplx 4
I JRAS03472_0151 Ceratosolen jucundus cplx 2
I JRAS03472_0155 Ceratosolen jucundus cplx 2
I JRAS03568_0251 Ceratosolen jucundus cplx 3
I JRAS03568_0252 Ceratosolen jucundus cplx 3
I JRAS01888_0151 Ceratosolen bisulcatus
S JRAS03568_0299 Ceratosolen jucundus cplx 3
S JRAS03568_0202 Ceratosolen jucundus cplx 3
S JRAS03568_0201 Ceratosolen jucundus cplx 3
I JRAS03568_0253 Ceratosolen jucundus cplx 3
I JRAS01888_0152 Ceratosolen bisulcatus
I JRAS02290_0156 Ceratosolen nsp047
S JRAS02290_0201 Ceratosolen nsp047
S JRAS02290_0202 Ceratosolen nsp047
S JRAS02290_0101 Ceratosolen nsp047
S JRAS02290_0102 Ceratosolen nsp047
I JRAS03568_0152 Ceratosolen nsp044
I JRAS03568_0153 Ceratosolen nsp044
S JRAS03568_0199 Ceratosolen nsp044
S JRAS03568_0101 Ceratosolen nsp044
I JRAS03568_0151 Ceratosolen nsp044
I JRAS03505_0156 Ceratosolen nsp035
I JRAS03505_0155 Ceratosolen nsp035
I JRAS03505_0151 Ceratosolen nsp035
S JRAS02409_0101 Ceratosolen wui ?
I JRAS02409_0156 Ceratosolen wui ?
I JRAS03590_0153 Ceratosolen cornutus
I JRAS02409_0155 Ceratosolen wui ?

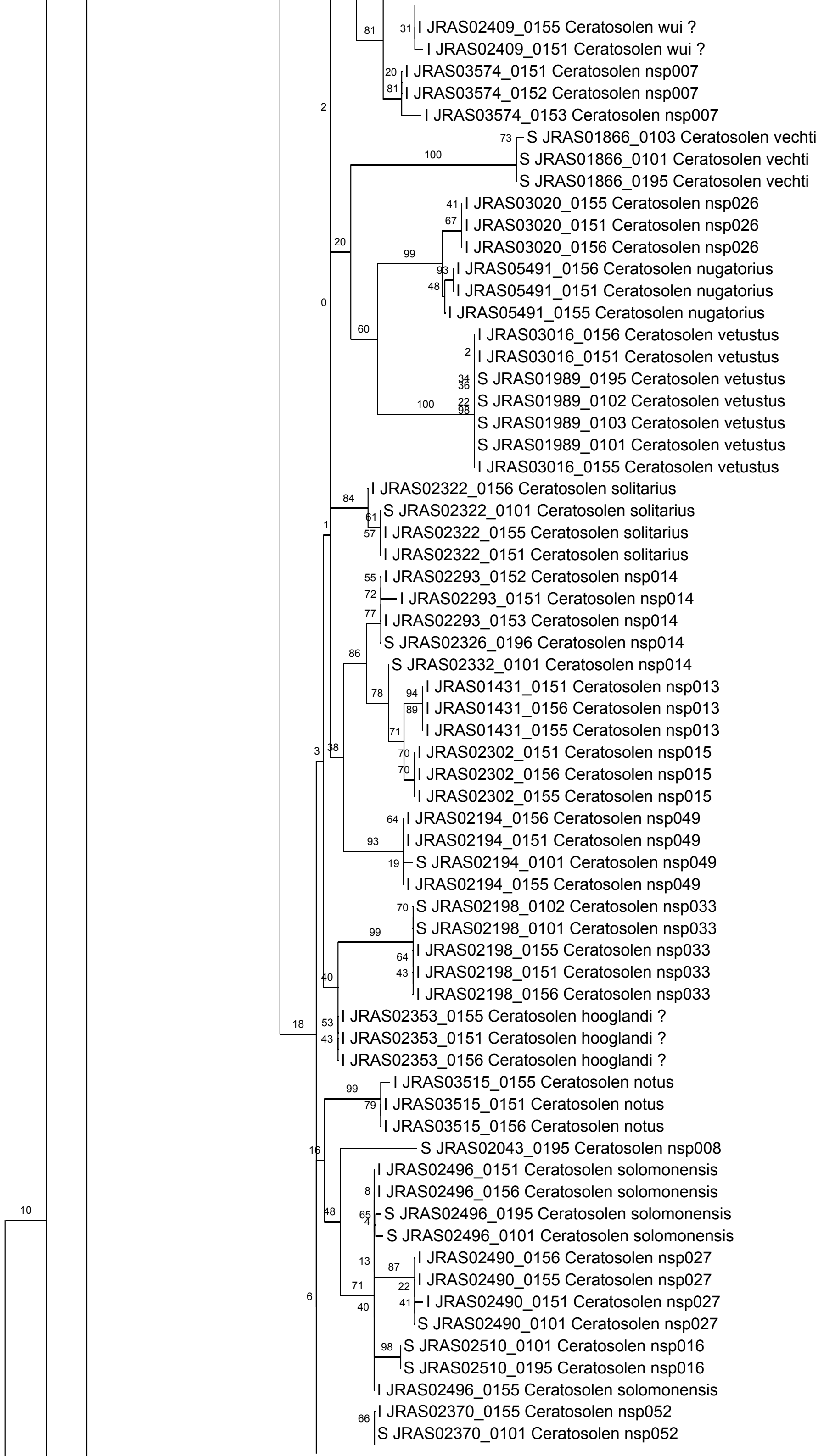
I JRAS02409_0155 Ceratosolen wui ?
I JRAS02409_0151 Ceratosolen wui ?
I JRAS03574_0151 Ceratosolen nsp007
I JRAS03574_0152 Ceratosolen nsp007
I JRAS03574_0153 Ceratosolen nsp007
S JRAS01866_0103 Ceratosolen vechti
S JRAS01866_0101 Ceratosolen vechti
S JRAS01866_0195 Ceratosolen vechti
I JRAS03020_0155 Ceratosolen nsp026
I JRAS03020_0151 Ceratosolen nsp026
I JRAS03020_0156 Ceratosolen nsp026
I JRAS05491_0156 Ceratosolen nugatorius
I JRAS05491_0151 Ceratosolen nugatorius
I JRAS05491_0155 Ceratosolen nugatorius
I JRAS03016_0156 Ceratosolen vetustus
I JRAS03016_0151 Ceratosolen vetustus
S JRAS01989_0195 Ceratosolen vetustus
S JRAS01989_0102 Ceratosolen vetustus
S JRAS01989_0103 Ceratosolen vetustus
S JRAS01989_0101 Ceratosolen vetustus
I JRAS03016_0155 Ceratosolen vetustus
I JRAS02322_0156 Ceratosolen solitarius
S JRAS02322_0101 Ceratosolen solitarius
I JRAS02322_0155 Ceratosolen solitarius
I JRAS02322_0151 Ceratosolen solitarius
I JRAS02293_0152 Ceratosolen nsp014
I JRAS02293_0151 Ceratosolen nsp014
I JRAS02293_0153 Ceratosolen nsp014
S JRAS02326_0196 Ceratosolen nsp014
S JRAS02332_0101 Ceratosolen nsp014
I JRAS01431_0151 Ceratosolen nsp013
I JRAS01431_0156 Ceratosolen nsp013
I JRAS01431_0155 Ceratosolen nsp013
I JRAS02302_0151 Ceratosolen nsp015
I JRAS02302_0156 Ceratosolen nsp015
I JRAS02302_0155 Ceratosolen nsp015
I JRAS02194_0156 Ceratosolen nsp049
I JRAS02194_0151 Ceratosolen nsp049
S JRAS02194_0101 Ceratosolen nsp049
I JRAS02194_0155 Ceratosolen nsp049
S JRAS02198_0102 Ceratosolen nsp033
S JRAS02198_0101 Ceratosolen nsp033
I JRAS02198_0155 Ceratosolen nsp033
I JRAS02198_0151 Ceratosolen nsp033
I JRAS02198_0156 Ceratosolen nsp033
I JRAS02353_0155 Ceratosolen hooglandi ?
I JRAS02353_0151 Ceratosolen hooglandi ?
I JRAS02353_0156 Ceratosolen hooglandi ?
I JRAS03515_0155 Ceratosolen notus
I JRAS03515_0151 Ceratosolen notus
I JRAS03515_0156 Ceratosolen notus
S JRAS02043_0195 Ceratosolen nsp008
I JRAS02496_0151 Ceratosolen solomonensis
I JRAS02496_0156 Ceratosolen solomonensis
S JRAS02496_0195 Ceratosolen solomonensis
S JRAS02496_0101 Ceratosolen solomonensis
I JRAS02490_0156 Ceratosolen nsp027
I JRAS02490_0155 Ceratosolen nsp027
I JRAS02490_0151 Ceratosolen nsp027
S JRAS02490_0101 Ceratosolen nsp027
S JRAS02510_0101 Ceratosolen nsp016
S JRAS02510_0195 Ceratosolen nsp016
I JRAS02496_0155 Ceratosolen solomonensis
I JRAS02370_0155 Ceratosolen nsp052
S JRAS02370_0101 Ceratosolen nsp052

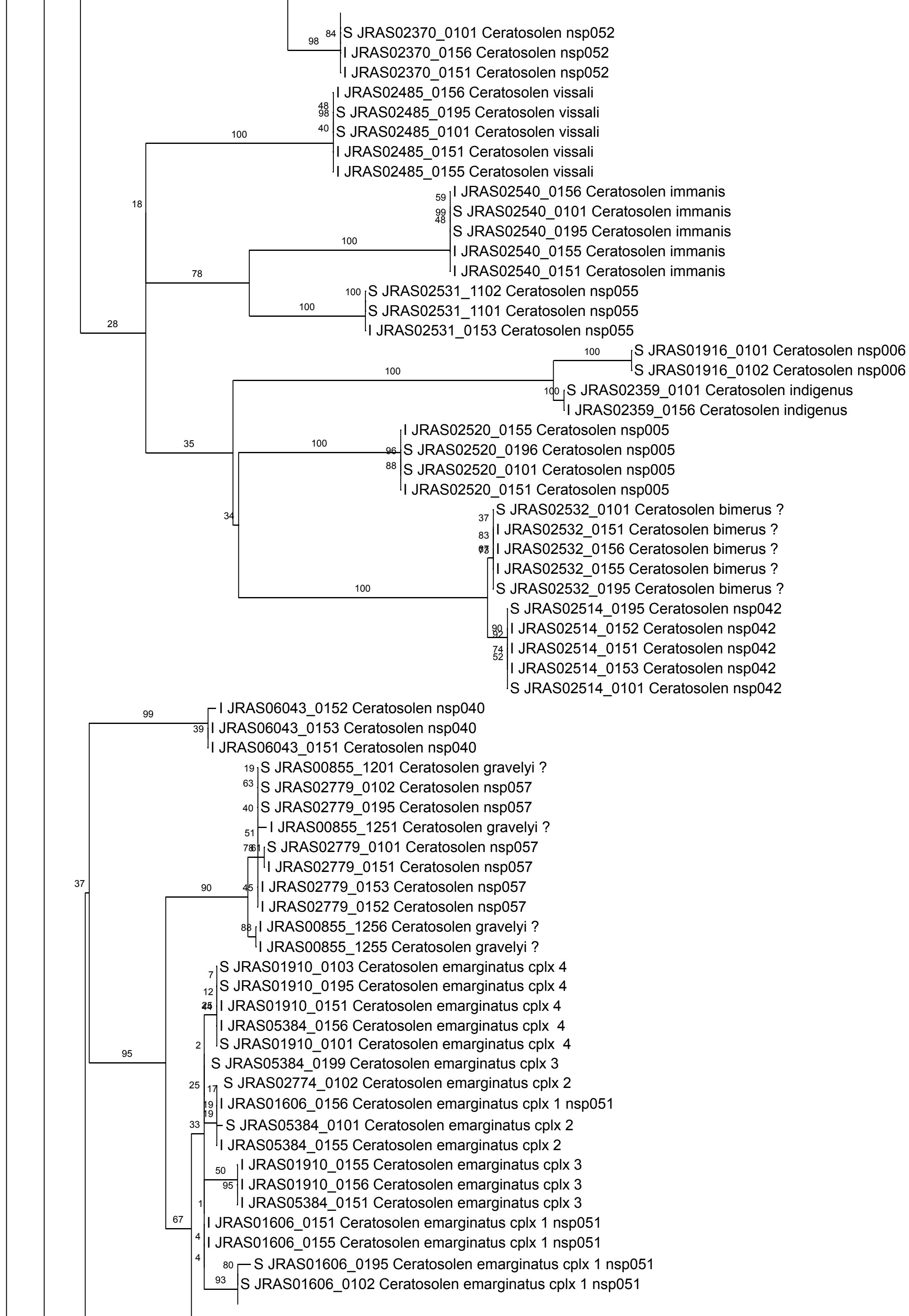
S JRAS02370_0101 Ceratosolen nsp052
I JRAS02370_0156 Ceratosolen nsp052
I JRAS02370_0151 Ceratosolen nsp052
I JRAS02485_0156 Ceratosolen vissali
S JRAS02485_0195 Ceratosolen vissali
S JRAS02485_0101 Ceratosolen vissali
I JRAS02485_0151 Ceratosolen vissali
I JRAS02485_0155 Ceratosolen vissali
I JRAS02540_0156 Ceratosolen immanis
S JRAS02540_0101 Ceratosolen immanis
S JRAS02540_0195 Ceratosolen immanis
I JRAS02540_0155 Ceratosolen immanis
I JRAS02540_0151 Ceratosolen immanis
S JRAS02531_1102 Ceratosolen nsp055
S JRAS02531_1101 Ceratosolen nsp055
I JRAS02531_0153 Ceratosolen nsp055
S JRAS01916_0101 Ceratosolen nsp006
S JRAS01916_0102 Ceratosolen nsp006
S JRAS02359_0101 Ceratosolen indigenus
I JRAS02359_0156 Ceratosolen indigenus
I JRAS02520_0155 Ceratosolen nsp005
S JRAS02520_0196 Ceratosolen nsp005
S JRAS02520_0101 Ceratosolen nsp005
I JRAS02520_0151 Ceratosolen nsp005
S JRAS02532_0101 Ceratosolen bimerus ?
I JRAS02532_0151 Ceratosolen bimerus ?
I JRAS02532_0156 Ceratosolen bimerus ?
I JRAS02532_0155 Ceratosolen bimerus ?
S JRAS02532_0195 Ceratosolen bimerus ?
S JRAS02514_0195 Ceratosolen nsp042
I JRAS02514_0152 Ceratosolen nsp042
I JRAS02514_0151 Ceratosolen nsp042
I JRAS02514_0153 Ceratosolen nsp042
S JRAS02514_0101 Ceratosolen nsp042
I JRAS06043_0152 Ceratosolen nsp040
I JRAS06043_0153 Ceratosolen nsp040
I JRAS06043_0151 Ceratosolen nsp040
S JRAS00855_1201 Ceratosolen gravelyi ?
S JRAS02779_0102 Ceratosolen nsp057
S JRAS02779_0195 Ceratosolen nsp057
I JRAS00855_1251 Ceratosolen gravelyi ?
S JRAS02779_0101 Ceratosolen nsp057
I JRAS02779_0151 Ceratosolen nsp057
I JRAS02779_0153 Ceratosolen nsp057
I JRAS02779_0152 Ceratosolen nsp057
I JRAS00855_1256 Ceratosolen gravelyi ?
I JRAS00855_1255 Ceratosolen gravelyi ?
S JRAS01910_0103 Ceratosolen emarginatus cplx 4
S JRAS01910_0195 Ceratosolen emarginatus cplx 4
I JRAS01910_0151 Ceratosolen emarginatus cplx 4
I JRAS05384_0156 Ceratosolen emarginatus cplx 4
S JRAS01910_0101 Ceratosolen emarginatus cplx 4
S JRAS05384_0199 Ceratosolen emarginatus cplx 3
S JRAS02774_0102 Ceratosolen emarginatus cplx 2
I JRAS01606_0156 Ceratosolen emarginatus cplx 1 nsp051
S JRAS05384_0101 Ceratosolen emarginatus cplx 2
I JRAS05384_0155 Ceratosolen emarginatus cplx 2
I JRAS01910_0155 Ceratosolen emarginatus cplx 3
I JRAS01910_0156 Ceratosolen emarginatus cplx 3
I JRAS05384_0151 Ceratosolen emarginatus cplx 3
I JRAS01606_0151 Ceratosolen emarginatus cplx 1 nsp051
I JRAS01606_0155 Ceratosolen emarginatus cplx 1 nsp051
S JRAS01606_0195 Ceratosolen emarginatus cplx 1 nsp051
S JRAS01606_0102 Ceratosolen emarginatus cplx 1 nsp051

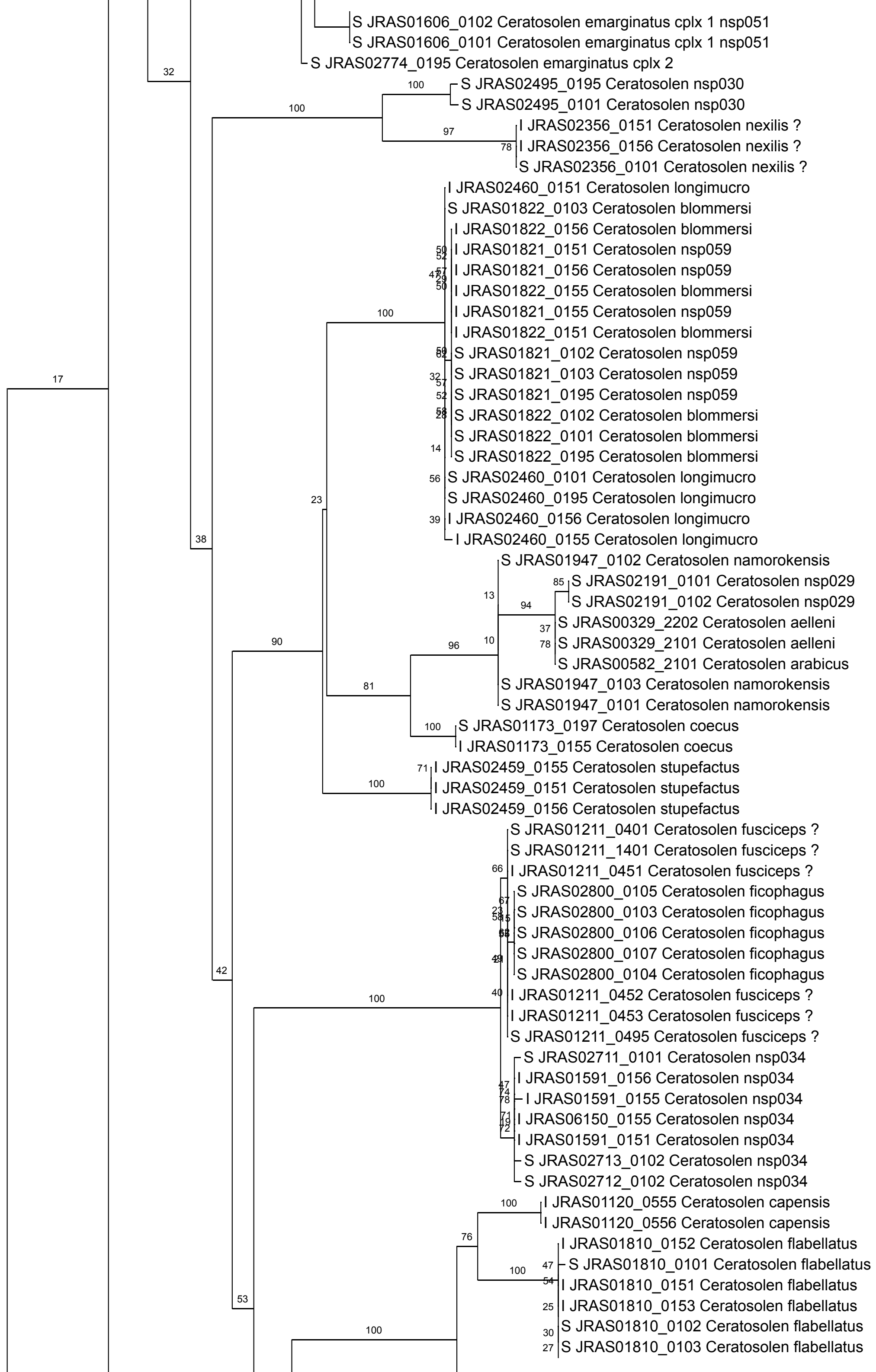

S JRAS01606_0102 Ceratosolen emarginatus cplx 1 nsp051
S JRAS01606_0101 Ceratosolen emarginatus cplx 1 nsp051
S JRAS02774_0195 Ceratosolen emarginatus cplx 2
S JRAS02495_0195 Ceratosolen nsp030
S JRAS02495_0101 Ceratosolen nsp030
I JRAS02356_0151 Ceratosolen nexilis ?
I JRAS02356_0156 Ceratosolen nexilis ?
S JRAS02356_0101 Ceratosolen nexilis ?
I JRAS02460_0151 Ceratosolen longimucro
S JRAS01822_0103 Ceratosolen blommersi
I JRAS01822_0156 Ceratosolen blommersi
I JRAS01821_0151 Ceratosolen nsp059
I JRAS01821_0156 Ceratosolen nsp059
I JRAS01822_0155 Ceratosolen blommersi
I JRAS01821_0155 Ceratosolen nsp059
I JRAS01822_0151 Ceratosolen blommersi
S JRAS01821_0102 Ceratosolen nsp059
S JRAS01821_0103 Ceratosolen nsp059
S JRAS01821_0195 Ceratosolen nsp059
S JRAS01822_0102 Ceratosolen blommersi
S JRAS01822_0101 Ceratosolen blommersi
S JRAS01822_0195 Ceratosolen blommersi
S JRAS02460_0101 Ceratosolen longimucro
S JRAS02460_0195 Ceratosolen longimucro
I JRAS02460_0156 Ceratosolen longimucro
I JRAS02460_0155 Ceratosolen longimucro
S JRAS01947_0102 Ceratosolen namorokensis
S JRAS02191_0101 Ceratosolen nsp029
S JRAS02191_0102 Ceratosolen nsp029
S JRAS00329_2202 Ceratosolen aelleni
S JRAS00329_2101 Ceratosolen aelleni
S JRAS00582_2101 Ceratosolen arabicus
S JRAS01947_0103 Ceratosolen namorokensis
S JRAS01947_0101 Ceratosolen namorokensis
S JRAS01173_0197 Ceratosolen coecus
I JRAS01173_0155 Ceratosolen coecus
I JRAS02459_0155 Ceratosolen stupefactus
I JRAS02459_0151 Ceratosolen stupefactus
I JRAS02459_0156 Ceratosolen stupefactus
S JRAS01211_0401 Ceratosolen fusciceps ?
S JRAS01211_1401 Ceratosolen fusciceps ?
I JRAS01211_0451 Ceratosolen fusciceps ?
S JRAS02800_0105 Ceratosolen ficophagus
S JRAS02800_0103 Ceratosolen ficophagus
S JRAS02800_0106 Ceratosolen ficophagus
S JRAS02800_0107 Ceratosolen ficophagus
S JRAS02800_0104 Ceratosolen ficophagus
I JRAS01211_0452 Ceratosolen fusciceps ?
I JRAS01211_0453 Ceratosolen fusciceps ?
S JRAS01211_0495 Ceratosolen fusciceps ?
S JRAS02711_0101 Ceratosolen nsp034
I JRAS01591_0156 Ceratosolen nsp034
I JRAS01591_0155 Ceratosolen nsp034
I JRAS06150_0155 Ceratosolen nsp034
I JRAS01591_0151 Ceratosolen nsp034
S JRAS02713_0102 Ceratosolen nsp034
S JRAS02712_0102 Ceratosolen nsp034
I JRAS01120_0555 Ceratosolen capensis
I JRAS01120_0556 Ceratosolen capensis
I JRAS01810_0152 Ceratosolen flabellatus
S JRAS01810_0101 Ceratosolen flabellatus
I JRAS01810_0151 Ceratosolen flabellatus
I JRAS01810_0153 Ceratosolen flabellatus
S JRAS01810_0102 Ceratosolen flabellatus
S JRAS01810_0103 Ceratosolen flabellatus

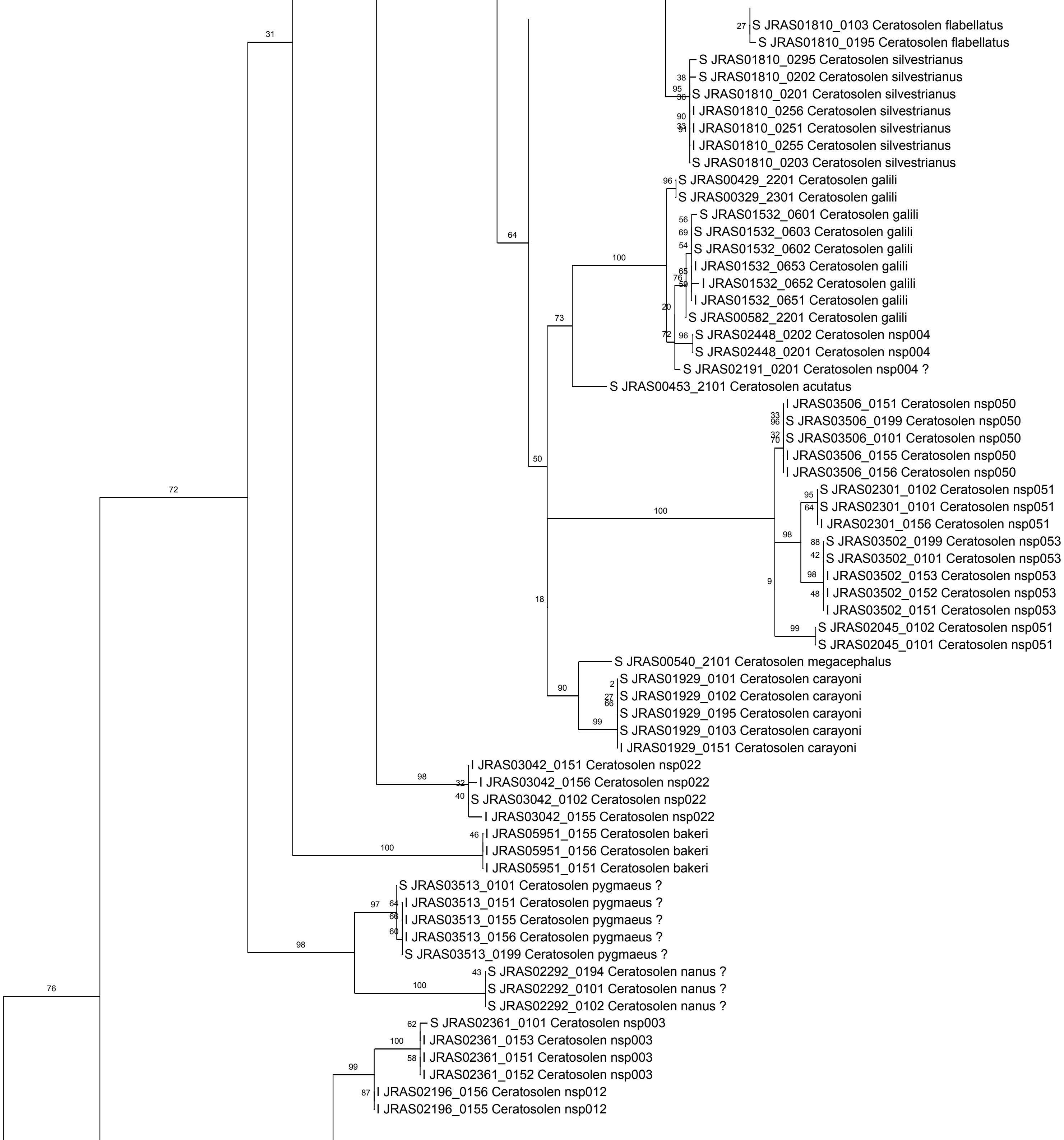
S JRAS01810_0103 Ceratosolen flabellatus
S JRAS01810_0195 Ceratosolen flabellatus
S JRAS01810_0295 Ceratosolen silvestrianus
S JRAS01810_0202 Ceratosolen silvestrianus
S JRAS01810_0201 Ceratosolen silvestrianus
I JRAS01810_0256 Ceratosolen silvestrianus
I JRAS01810_0251 Ceratosolen silvestrianus
I JRAS01810_0255 Ceratosolen silvestrianus
S JRAS01810_0203 Ceratosolen silvestrianus
S JRAS00429_2201 Ceratosolen galili
S JRAS00329_2301 Ceratosolen galili
S JRAS01532_0601 Ceratosolen galili
S JRAS01532_0603 Ceratosolen galili
S JRAS01532_0602 Ceratosolen galili
I JRAS01532_0653 Ceratosolen galili
I JRAS01532_0652 Ceratosolen galili
I JRAS01532_0651 Ceratosolen galili
S JRAS00582_2201 Ceratosolen galili
S JRAS02448_0202 Ceratosolen nsp004
S JRAS02448_0201 Ceratosolen nsp004
S JRAS02191_0201 Ceratosolen nsp004 ?
S JRAS00453_2101 Ceratosolen acutatus
I JRAS03506_0151 Ceratosolen nsp050
S JRAS03506_0199 Ceratosolen nsp050
S JRAS03506_0101 Ceratosolen nsp050
I JRAS03506_0155 Ceratosolen nsp050
I JRAS03506_0156 Ceratosolen nsp050
S JRAS02301_0102 Ceratosolen nsp051
S JRAS02301_0101 Ceratosolen nsp051
I JRAS02301_0156 Ceratosolen nsp051
S JRAS03502_0199 Ceratosolen nsp053
S JRAS03502_0101 Ceratosolen nsp053
I JRAS03502_0153 Ceratosolen nsp053
I JRAS03502_0152 Ceratosolen nsp053
I JRAS03502_0151 Ceratosolen nsp053
S JRAS02045_0102 Ceratosolen nsp051
S JRAS02045_0101 Ceratosolen nsp051
S JRAS00540_2101 Ceratosolen megacephalus
S JRAS01929_0101 Ceratosolen carayoni
S JRAS01929_0102 Ceratosolen carayoni
S JRAS01929_0195 Ceratosolen carayoni
S JRAS01929_0103 Ceratosolen carayoni
I JRAS01929_0151 Ceratosolen carayoni
I JRAS03042_0151 Ceratosolen nsp022
I JRAS03042_0156 Ceratosolen nsp022
S JRAS03042_0102 Ceratosolen nsp022
I JRAS03042_0155 Ceratosolen nsp022
I JRAS05951_0155 Ceratosolen bakeri
I JRAS05951_0156 Ceratosolen bakeri
I JRAS05951_0151 Ceratosolen bakeri
S JRAS03513_0101 Ceratosolen pygmaeus ?
I JRAS03513_0151 Ceratosolen pygmaeus ?
I JRAS03513_0155 Ceratosolen pygmaeus ?
I JRAS03513_0156 Ceratosolen pygmaeus ?
S JRAS03513_0199 Ceratosolen pygmaeus ?
S JRAS02292_0194 Ceratosolen nanus ?
S JRAS02292_0101 Ceratosolen nanus ?
S JRAS02292_0102 Ceratosolen nanus ?
S JRAS02361_0101 Ceratosolen nsp003
I JRAS02361_0153 Ceratosolen nsp003
I JRAS02361_0151 Ceratosolen nsp003
I JRAS02361_0152 Ceratosolen nsp003
I JRAS02196_0156 Ceratosolen nsp012
I JRAS02196_0155 Ceratosolen nsp012

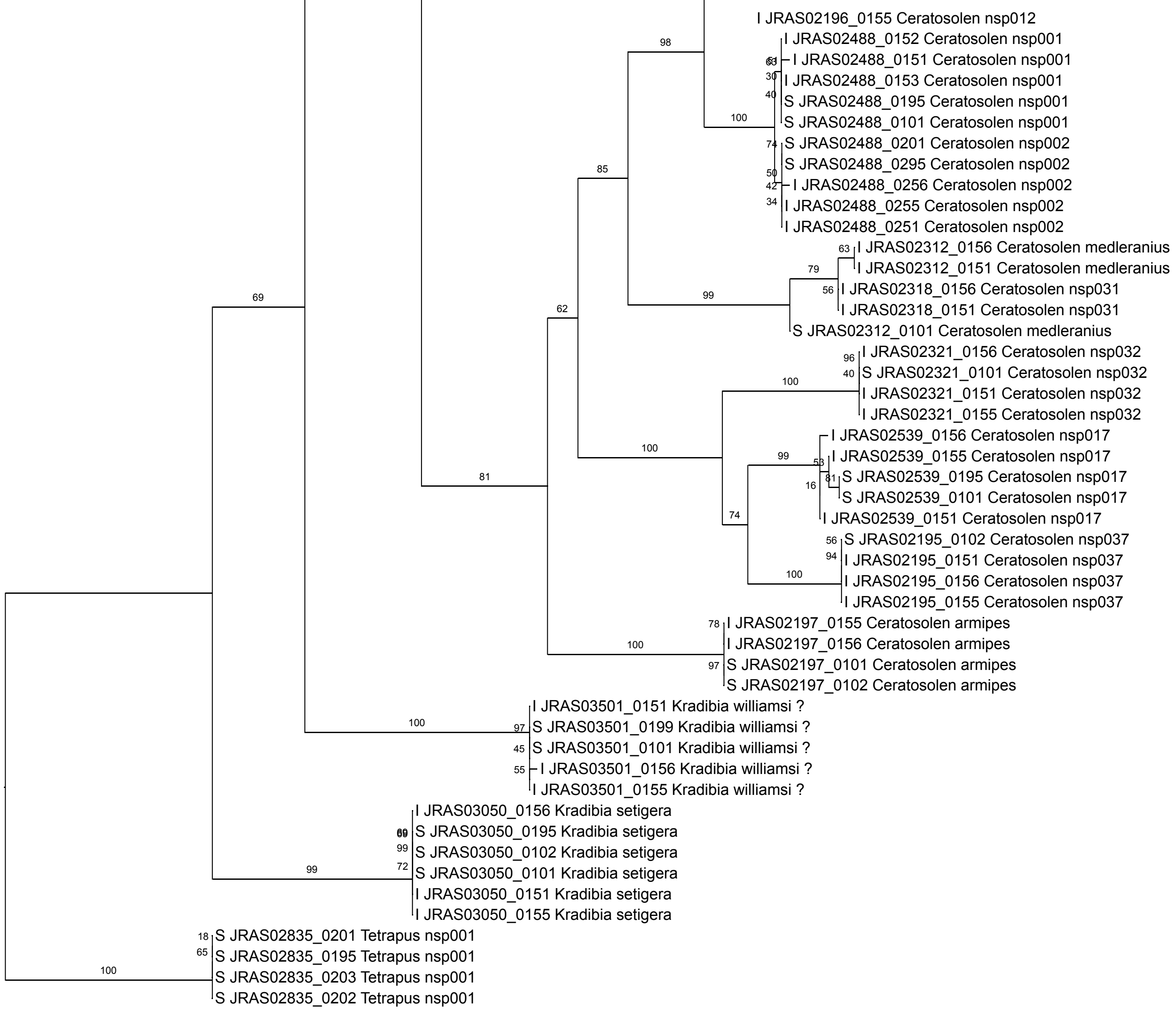
I JRAS02196_0155 Ceratosolen nsp012
I JRAS02488_0152 Ceratosolen nsp001
I JRAS02488_0151 Ceratosolen nsp001
I JRAS02488_0153 Ceratosolen nsp001
S JRAS02488_0195 Ceratosolen nsp001
S JRAS02488_0101 Ceratosolen nsp001
S JRAS02488_0201 Ceratosolen nsp002
S JRAS02488_0295 Ceratosolen nsp002
I JRAS02488_0256 Ceratosolen nsp002
I JRAS02488_0255 Ceratosolen nsp002
I JRAS02488_0251 Ceratosolen nsp002
I JRAS02312_0156 Ceratosolen medleranius
I JRAS02312_0151 Ceratosolen medleranius
I JRAS02318_0156 Ceratosolen nsp031
I JRAS02318_0151 Ceratosolen nsp031
S JRAS02312_0101 Ceratosolen medleranius
I JRAS02321_0156 Ceratosolen nsp032
S JRAS02321_0101 Ceratosolen nsp032
I JRAS02321_0151 Ceratosolen nsp032
I JRAS02321_0155 Ceratosolen nsp032
I JRAS02539_0156 Ceratosolen nsp017
I JRAS02539_0155 Ceratosolen nsp017
S JRAS02539_0195 Ceratosolen nsp017
S JRAS02539_0101 Ceratosolen nsp017
I JRAS02539_0151 Ceratosolen nsp017
S JRAS02195_0102 Ceratosolen nsp037
I JRAS02195_0151 Ceratosolen nsp037
I JRAS02195_0156 Ceratosolen nsp037
I JRAS02195_0155 Ceratosolen nsp037
I JRAS02197_0155 Ceratosolen armipes
I JRAS02197_0156 Ceratosolen armipes
S JRAS02197_0101 Ceratosolen armipes
S JRAS02197_0102 Ceratosolen armipes
I JRAS03501_0151 Kradibia williamsi ?
S JRAS03501_0199 Kradibia williamsi ?
S JRAS03501_0101 Kradibia williamsi ?
I JRAS03501_0156 Kradibia williamsi ?
I JRAS03501_0155 Kradibia williamsi ?
I JRAS03050_0156 Kradibia setigera
S JRAS03050_0195 Kradibia setigera
S JRAS03050_0102 Kradibia setigera
S JRAS03050_0101 Kradibia setigera
I JRAS03050_0151 Kradibia setigera
I JRAS03050_0155 Kradibia setigera
S JRAS02835_0201 Tetrapus nsp001
S JRAS02835_0195 Tetrapus nsp001
S JRAS02835_0203 Tetrapus nsp001
S JRAS02835_0202 Tetrapus nsp001
